# DAZL regulates proliferation of human primordial germ cells by direct binding to precursor miRNAs and enhances DICER processing activity

**DOI:** 10.1093/nar/gkac856

**Published:** 2022-10-24

**Authors:** An Yan, Jie Xiong, Jiadong Zhu, Xiangyu Li, Shuting Xu, Xiaoyu Feng, Xin Ke, Zhenyi Wang, Yang Chen, Hong-Wei Wang, Michael Q Zhang, Kehkooi Kee

**Affiliations:** Center for Stem Cell Biology and Regenerative Medicine, Department of Basic Medical Sciences, School of Medicine, Tsinghua University, Beijing 100084, China; Center for Stem Cell Biology and Regenerative Medicine, Department of Basic Medical Sciences, School of Medicine, Tsinghua University, Beijing 100084, China; Tsinghua University-–Peking University Joint Center for Life Sciences, Tsinghua University, Beijing 100084, China; Center for Stem Cell Biology and Regenerative Medicine, Department of Basic Medical Sciences, School of Medicine, Tsinghua University, Beijing 100084, China; School of Software Engineering, Beijing Jiaotong University, Beijing 100044, China; MOE Key Laboratory of Bioinformatics; Bioinformatics Division and Center for Synthetic & Systems Biology, BNRist; Department of Automation, Tsinghua University, Beijing 100084, China; Center for Stem Cell Biology and Regenerative Medicine, Department of Basic Medical Sciences, School of Medicine, Tsinghua University, Beijing 100084, China; Tsinghua University-–Peking University Joint Center for Life Sciences, Tsinghua University, Beijing 100084, China; Center for Stem Cell Biology and Regenerative Medicine, Department of Basic Medical Sciences, School of Medicine, Tsinghua University, Beijing 100084, China; Ministry of Education Key Laboratory of Protein Sciences, Tsinghua–Peking Joint Center for Life Sciences, Beijing Advanced Innovation Center for Structural Biology, School of Life Sciences, Tsinghua University, Beijing 100084,China; MOE Key Laboratory of Bioinformatics; Bioinformatics Division and Center for Synthetic & Systems Biology, BNRist; Department of Automation, Tsinghua University, Beijing 100084, China; MOE Key Laboratory of Bioinformatics; Bioinformatics Division and Center for Synthetic & Systems Biology, BNRist; Department of Automation, Tsinghua University, Beijing 100084, China; School of Medicine, Tsinghua University, Beijing 100084, China; Ministry of Education Key Laboratory of Protein Sciences, Tsinghua–Peking Joint Center for Life Sciences, Beijing Advanced Innovation Center for Structural Biology, School of Life Sciences, Tsinghua University, Beijing 100084,China; MOE Key Laboratory of Bioinformatics; Bioinformatics Division and Center for Synthetic & Systems Biology, BNRist; Department of Automation, Tsinghua University, Beijing 100084, China; School of Medicine, Tsinghua University, Beijing 100084, China; Department of Biological Sciences, Center for Systems Biology, The University of Texas at Dallas, 800 West Campbell Road, RL11, Richardson, TX 75080-3021, USA; Center for Stem Cell Biology and Regenerative Medicine, Department of Basic Medical Sciences, School of Medicine, Tsinghua University, Beijing 100084, China; Tsinghua University-–Peking University Joint Center for Life Sciences, Tsinghua University, Beijing 100084, China

## Abstract

Understanding the molecular and cellular mechanisms of human primordial germ cells (hPGCs) is essential in studying infertility and germ cell tumorigenesis. Many RNA-binding proteins (RBPs) and non-coding RNAs are specifically expressed and functional during hPGC developments. However, the roles and regulatory mechanisms of these RBPs and non-coding RNAs, such as microRNAs (miRNAs), in hPGCs remain elusive. In this study, we reported a new regulatory function of DAZL, a germ cell-specific RBP, in miRNA biogenesis and cell proliferation. First, DAZL co-localized with miRNA let-7a in human PGCs and up-regulated the levels of >100 mature miRNAs, including eight out of nine let-7 family, miR21, miR22, miR125, miR10 and miR199. Purified DAZL directly bound to the loops of precursor miRNAs with sequence specificity of GUU. The binding of DAZL to the precursor miRNA increased the maturation of miRNA by enhancing the cleavage activity of DICER. Furthermore, cell proliferation assay and cell cycle analysis confirmed that DAZL inhibited the proliferation of *in vitro* PGCs by promoting the maturation of these miRNAs. Evidently, the mature miRNAs up-regulated by DAZL silenced cell proliferation regulators including TRIM71. Moreover, DAZL inhibited germline tumor cell proliferation and teratoma formation. These results demonstrate that DAZL regulates hPGC proliferation by enhancing miRNA processing.

## INTRODUCTION

Human primordial germ cells (hPGCs) are derived from pluripotent epiblasts and further develop into gametes after completing meiosis (1). Therefore, hPGCs undergo transition from a pluripotent and proliferative state into a unipotent and quiescent state. Female hPGCs exit the mitotic cycle and enter meiosis after 11–14 weeks of gestation, whereas male hPGCs enter mitotic arrest around the same period (1). The irregular proliferation and persistent pluripotency of hPGCs have been linked to testicular tumors and ovarian cancers (2,3). However, the factors and mechanisms regulating the proliferation of hPGCs during this transition are unclear.

DAZL, an RNA-binding protein (RBP), is essential in ensuring germ cell development in mice (4–9). Dazl-deficient mice show defects in germ cell production in both male and female gonads because of incomplete meiosis. Recent studies have shown that DAZL deletion causes spontaneous gonadal teratomas in mice and pigs (10), whereas an overexpression of DAZL in hPGC-like cells (hPGCLCs) reduces the expression of pluripotency markers, including OCT4 and NANOG (11). These results indicate that DAZL is a key determinant of mammalian PGCs. At the molecular level, DAZL binds to many mRNA transcripts in the 3′-untranslated region (UTR) and functions as a repressor or activator of translation during both spermatogenesis and oocyte maturation (12–14). DAZL has a highly conserved domain, the RNA recognition motif (RRM), that can bind targeted RNAs (15). The RRM domain, which can recognize GUU triplets in different sequence contexts, is necessary for RNA binding (12,16–18). Several molecular studies of DAZL have focused on the regulatory roles of the coding genes, but no study has reported its potential role in the regulation of non-coding genes, such as microRNAs (miRNAs) during PGC development.

Recently, growing evidence has supported the essential role of miRNAs in gametogenesis and fertility (19). In PGCs, several miRNAs are highly expressed and regulate distinct cellular pathways that determine germ cell development (20,21). The biogenesis of miRNAs can be regulated at several levels, including primary miRNA (pri-miRNA) transcription, pri-miRNA processing via DROSHA and precursor miRNA (pre-miRNA) processing via DICER (22,23). Moreover, DICER processing is regulated by several cofactor proteins, including the HIV-1 TAR RBP (TRBP), the protein activator of protein kinase R (PACT), and adenosine deaminases acting on RNA (ADARs). They play crucial roles in substrate selection, determination of cleavage sites of pre-miRNAs and the dicing activity (24–31). These results indicate that RBPs are essential regulators of miRNA functions. However, germ cell-specific RBPs have not been reported in miRNA biogenesis and functions.

In this study, we demonstrate that DAZL directly binds to pre-miRNAs and enhances the processing of pre-miRNAs to mature miRNAs, thereby reducing the proliferation of hPGCs and teratoma formation.

## MATERIALS AND METHODS

### Animal care and use

BALB/c nude mice were purchased from Vital River Laboratory Animal Technology Co., Ltd (Beijing, China). In all experiments, 8-week-old female mice were used. All animal maintenance and experimental procedures were performed according to the guidelines of the Institutional Animal Care and Use Committee (IACUC) of Tsinghua University, Beijing, China.

### Cell lines

hESC line H9 (female line) was obtained from WiCell, Inc., and HSF6 (female line) was a gift from Renee Reijo Pera's laboratory. These hESCs were maintained on inactivated mouse embryo fibroblasts (MEFs), cultured in 8 ng/ml basic fibroblast growth factor (FGF; R&D systems) in addition to 80% KnockOut™ Dulbecco’s modified Eagle’s medium (DMEM; 10829018, Gibco), 20% KnockOut™ Serum Replacement (10828028, Gibco), 1 mM GlutaMAX™ Supplement (35050061, Gibco), 0.1 mM MEM non-essential amino acids (11140050, Gibco) and 100 U/ml penicillin–streptomycin (30-002-CI, Corning).

TGCT-derived N-tera2 (NT2) cells were a gift from Peter Andrew's laboratory and were cultured in 90% DMEM (10-017-CV, Corning), 10% fetal bovine serum (FBS (#900-108, GEMINI), 1 mM GlutaMAX, 0.1 mM MEM non-essential amino acids and 100 U/ml penicillin–streptomycin. 293FT (human female cell line) was maintained in 90% DMEM, 10% FBS, 1 mM GlutaMAX, 0.1 mM MEM non-essential amino acids, 1 mM sodium pyruvate, 0.5 mg/ml geneticin and 100 U/ml penicillin–streptomycin.

All the cell lines were cultured at 37°C in a humid atmosphere with 5% CO_2_.

### Tissue samples

Human fetal ovaries were procured with the approval of the Institutional Review Board (IRB) of the Tsinghua University Second Hospital and the IRB of the School of Medicine, Tsinghua University. All fetal ovaries were collected from elected abortions with consent forms, and the donor information was kept confidential from the researchers. All tissues were only used for histological analysis in this project without any further expansion or derivations of living cells.

### Lentiviral production

Lentivirus was produced by transfection of lentivectors together with Δ8.9 and Vsvg plasmids using Lipofectamine 2000 (11668027, Invitrogen) in 293FT cells at 70–90% confluency as previously described (32). At 6 h after transfection, medium was replaced with fresh 293FT medium without antibiotics, and supernatant was collected after 72 h culture in a cell incubator.

### Silencing (shRNA) of DAZL

All oligodeoxyribonucleotides used for the construction of small-hairpin RNA (shRNA) vectors were obtained from RuiBiotech (sequences are listed below). Control shRNA with a hairpin sequence of LacZ: acaaaaaaaaatcgctgatttgtgtagtctctcttgaagactacacaaatcagcgattt was employed as a negative control. Sequences of shRNA (sense strand, 5΄→3΄): shDAZL-4, CAGAAGATAGTAGAATCAC; shDAZL-6, GTGGTATCTTGTCTGTTTA were as previously described (32). hESCs were infected with lentivirus expressing shDAZL4–red fluorescent protein (RFP), shDAZL6–RFP or shLacZ–RFP (control cells) at 50% confluency, followed by differentiation to hPGCLCs for 6 days before fluorescence-activated cell sorting (FACS) to isolated RFP-positive hPGCLCs for further analysis.

### Immunofluorescence (IF) of fetal ovary sections

Paraffin sections were subjected to antigen repair after the dewaxing and rehydration steps were completed. Sections were washed in Tris-buffered saline (TBS) with 0.1% Triton X-100 for 5 min and blocked with 10% goat serum in TBS for 1 h at room temperature. Blocked sections were then incubated overnight at 4°C with primary antibody [1:50 DAZL (MCA2336, Bio-Rad), 1:50 DICER (20567-1-AP, Proteintech), 1:250 KI-67 (mA5-14520, Thermo Fisher Scientific) and DGCR8 (25835-1-AP, Proteintech)]. Sections were rinsed with TBST for 5 min, three times and further incubated with secondary antibody (1:1000) for 1 h at room temperature. Sections were rinsed with TBST for 5 min twice and stained with 1 μg/ml 4′,6-diamidino-2-phenylindole (DAPI) for 10 min at room temperature. Slides were mounted with ProLong® Gold Antifade (P36934, Invitrogen).

### RNA FISH and IF co-staining

RNA fluorescence *in situ* hybridization (FISH) and IF assay was performed with digoxin-labeled cDNA let-7a-5p probes (Guangzhou EXON Biological Technology) using a previously described protocol (33). In brief, the samples were pre-hybridized for 20 min at 25°C below the predicted *T*_m_ of the probe, and then hybridization was performed for 1 h at the same temperature, followed by 3–5 washes with wash buffer. Cells were incubated with anti-digoxigenin (DIG) antibody (1:200, Guangzhou EXON Biological Technology D-2406B) overnight at 4°C. After RNA FISH, IF was performed to detect proteins. Briefly, cells were blocked with 2% FBS and 1% bovine serum albumin (BSA) in phosphate-buffered saline (PBS) for 1 h at room temperature and then incubated for 1 h at room temperature with primary antibody [DAZL (1:50) and LIN28A (1:100)]. The following steps of IF were performed as described above.

### Protein purification

Plasmids expressing proteins of interest fused with an N-terminal glutathione *S*-transferase (GST) tag were transformed into *Escherichia coli* BL21 strain. Expression was induced by addition of 50 mg/l isopropyl-β-d-thiogalactoside (IPTG) at 18°C to logarithmically growing cultures. Bacteria were harvested by centrifugation at 4°C, 4000 rpm for 15 min and pellets were resuspended by lysis buffer [20 mM Tris–HCl pH 8.0, 500 mM NaCl, 1 mM MgCl_2_, 1 mM phenylmethylsulfonyl fluoride (PMSF), 1 ng/ml DNase I]. Cells in lysis buffer were lysed by a high pressure homogenizer, and cell debris was removed by centrifugation at 4°C. The supernatant was loaded onto a glutathione column (Bio-Rad) and then washed by cell lysis buffer. The target proteins were eluted with elution buffer (20 mM Tris–HCl pH 8.0, 150 mM NaCl, 20 mM glutathione). Protein solutions were concentrated for storage with a 15 kDa cut-off ultrafiltration device (Millipore). The concentrated solution was subjected to a Source S column (GE Healthcare) with buffer A (50 mM Tris–HCl pH 6.3) and buffer B (50 mM Tris–HCl pH 6.3, 1 M NaCl). Target proteins were concentrated and finally purified by size-exclusion chromatography using a Superdex 75 10/300 GL column (GE Healthcare) in buffer containing 30 mM Tris–HCl pH 6.8, 50 mM NaCl, 2 mM MgCl_2_, 1 mM dithiothreitol (DTT). Purity of proteins was detected by Coomassie brilliant blue staining and western blot. All the above operations were executed in a 4°C cold room. Aliquots of proteins were stored at –80°C.

### Electrophoretic mobility shift assay (EMSA)

Pre-let-7 labeled with FAM at the 5′ terminus (10 nM) was incubated with different dosages of DAZL mentioned in the figure legends for 40 min in a final 10 μl reaction solution containing 10 mM HEPES (pH 7.4), 50 mM KCl, 1 mM EDTA, 0.05% Triton X-100, 5% glycerol, 0.01 mg/ml BSA and 1 mM DTT. The products were resolved through 1% agarose gels in 0.5× Tris-borate-EDTA buffer under an electric field of 15 V/cm for 30 min and the gels were subsequently visualized with a Typhoon Trio Imager (Amersham Biosciences).

### Fluorescence polarization assay (FP assay)

For 384-well FP assays, 10 nM pre-let-7 labeled with FAM at the 5′ terminus was incubated with different dosages of DAZL for 40 min in a final 20 μl reaction solution containing 10 mM HEPES (pH 7.4), 50 mM KCl, 1 mM EDTA, 0.05% Triton X-100, 5% glycerol, 0.01 mg/ml BSA and 1 mM DTT. The products were added on a 384-well plate (CLS3544-50EA, Corning low volume flat bottom polystyrene non-binding surface). Any bubbles should be avoided. FP measurements were taken on a EnVision Multimode Microplate Reader (Perkin).

### Dicing activity assay

Dicing activity assay was performed as previously described (27). Briefly, the pre-let-7 RNA (5′-6-FAM-UGAGGUAGUAGGUUGUAUAGUUUUAGGGUCACACCCACCACUGGGAGAUAACUAUACAAUCUACUGUCUUACC-3′, Genescript Inc.) and the pre-let-7 RNA modified in the loop region (5′-6-FAM-UGAGGUAGUAGGUUGUAUAGUUUUAGAGUUACACCCUGGGAGUUAACUAUACAAUCUACUGUCUUACC-3′, Genescript Inc.) were used in dicing the assay of Figure 4C–G and Figure 4D, respectively. The reaction buffer containing 30 mM Tris–HCl (pH 6.8), 50 mM NaCl, 2 mM MgCl_2_, 0.1% Triton X-100, 15 ± 25% glycerol and 1 mM DTT. We mixed 2 μl of 20 nM FAM-labeled pre-let-7 with 2 μl of 100 nM hDICER and different dosages of DAZL mentioned in the main text in a final 15 μl reaction solution, and incubated the solution at 37°C for 15min. The reaction was stopped with RNA loading buffer containing 95% formamide and 20 mM EDTA, boiled for 5 min and chilled on ice. RNA products were analyzed by 18% polyacrylamide, 8 M urea denaturing gel electrophoresis, and visualized with a Typhoon Trio Imager (Amersham Biosciences).

### Co-immunoprecipitation (co-IP)

For anti-DICER immunoprecipitation from 293FT cells, cells were transfected with oeDAZL plasmid for 48 h before lysis. The cellular extract was treated with 80 μg/ml RNase A and 200 U/ml RNase T1 (EN0551, Thermo Scientific) for 15 min at 37°C before IP. Lysates were incubated with anti-DICER antibody (20567-1-AP, Proteintech) at 4°C for 2 h. Then Dynabeads Protein G (10003D, Thermo Fisher Scientific) were added to the lysates and the lysates were incubated at 4°C for 2 h. Beads were then washed at least twice with PBS and 0.02% Tween-20. The beads–protein mixture was finally eluted with 2× protein loading buffer and analysed by western blotting.

### ES cell line differentiation

For differentiation of hESCs, cells were first seeded onto a 6-well-plate coated with Matrigel (354248, Corning) at 40–50% confluency and followed by 1 day recovery. These cells were then differentiated directly with 50 ng/ml bone morphogenetic protein 4 (BMP4) and 50 ng/ml BMP8a in differentiation medium (90% KO-DMEM, 10% FBS, 1 mM GlutaMAX, 0.1 mM MEM non-essential amino acids and 100 U/ml penicillin–streptomycin) for 6 days. For overexpression and silencing experiments, cells were treated with 50 ng/ml BMP4 and 50 ng/ml BMP8a in differentiation medium for 2 h, transduced with lentivirus and differentiated in differentiation medium with 50 ng/ml BMP4 and 50 ng/ml BMP8a for another 5 days. Medium was changed every 3 days. Cells were collected and analyzed by Influx (BD) on day 6.

### RNA immunoprecipitation

Before RNA IP (RIP), anti-V5 agarose affinity gel (A7345, Sigma-Aldrich) was blocked with 1 mg/ml BSA and 100 ng/ml yeast tRNA, and washed with chilled lysis buffer. A total of 1 × 10^6^ cells were collected, washed with chilled PBS and lysed in lysis buffer [50 mM pH 7.4 Tris–HCl, 5% glycerol, 1 mM EDTA, 1% Triton X-100, 150 mM NaCl, 0.1% sodium deoxycholate, 50 U/ml SUPERase•In™ (AM2694, Thermo Fisher Scientific), 1 × cOmplete™, EDTA-free Protease Inhibitor Cocktail (04693132001, Roche), 1 mM DTT, 1 mM PMSF (93482, Sigma-Aldrich)]. Cell lysate was then treated with DNase (#B0303S, NEB) at 37°C for 5 min and the supernatant was incubated with blocked anti-V5 agarose affinity gel at 4°C overnight. After incubation, anti-V5 agarose affinity gel was washed three times and treated with proteinase K (P8107S, NEB). RNA in the supernatant was extracted using TRIzol™ Reagent (15596018, Thermo Fisher Scientific).

### miRNA library preparation

The miRNA library was prepared using the NEBNext® Multiplex Small RNA Library Prep Set for Illumina according to the manufacturer's instruction. Cells were firstly seeded onto a 6-well plate coated with Matrigel (354248, Corning) at 40–50% confluency and followed by 1 day recovery. After 2 h treatment in differentiation medium (90% KO-DMEM, 10% FBS, NEAA, Glutamax, P/S) containing 50 ng/ml BMP4 and 50 ng/ml BMP8a, cells were transduced with lentivirus overexpressing DAZL–green fluorescent protein (GFP) or control GFP. Cells were then differentiated until day 6 for collection by FACS (Influx, BD). RNA was extracted using TRIzol™ Reagent (15596018, Thermo Fisher Scientific) and 1 μg of total RNA was used as starting material.

### EdU proliferation assay

5-Ethynyl-2′-deoxyuridine (EdU) proliferation assay was performed using the Cell-Light™ EdU Apollo® 643 In Vitro Flow Cytometry Kit or the BeyoClick™ EdU Cell Proliferation Kit with Alexa Fluor 647. Briefly, cells were infected with lentivirus expressing DAZL-P2A–GFP or GFP at 50% confluency. hESCs were differentiated until day 6 while NT2 was treated with 10 μg/ml blasticidin for 2 days and cultured until day 4. When Cell-Light™ EdU Apollo® 643 was used for the proliferation assay, cells were incubated with 50 μM EdU for 2 h, washed with PBS twice and collected in a 15 ml tube. Cells were fixed with 4% paraformaldehyde (PFA) for 15–30 min, neutralized with 2 mg/ml glycine for 5 min, washed once with PBS, treated with 0.5% Triton X-100 for 10 min and washed once with PBS. Cells were then stained with Apollo staining buffer for 30 min in the dark and washed with PBST three times. When the BeyoClick™ EdU Cell Proliferation Kit with Alexa Fluor 647 was used for the proliferation assay, cells were incubated with 10 μM EdU for 2 h at 37°C. After collection, cells were post-fixed with 4% PFA for 10 min, washed once with washing buffer (3% BSA in PBS), permeabilized with 0.3% Triton X-100 in PBS for 10 min and rinsed with washing buffer once. Thereafter, the cells were incubated with freshly prepared Click-iT EdU detection cocktail for 30 min at room temperature and rinsed with washing buffer twice. The proportion of cells which incorporated EdU was analyzed by flow cytometry (Influx/LSRFortessa, BD).

### Cell cycle analysis

Cells were infected with lentivirus expressing DAZL-P2A–GFP or GFP at 50% confluency. hESCs were differentiated until day 6 while NT2 was treated with 10 μg/ml blasticidin for 2 days and cultured until day 4. Cells were then stained with 5 μg/ml Hoechst 33342 (C1026, Beyotime) and 20 μM varapamil hydrochloride (V4629, Sigma-Aldrich) at 37°C for 1 h, washed with PBS once and collected for analysis by flow cytometry (LSRFortessa, BD).

### Teratoma assay and H&E staining in nude mice

NT2 cells were infected with lentivirus expressing oeDAZL (DAZL-P2A–GFP) or control (GFP), and GFP+ cells were isolated by flow cytometry and suspended in 50% Matrigel (354248, Corning). Eight-week-old BALB/c nude mice were anesthetized with 240 mg/kg avertin (T4840-2, Sigma-Aldrich) and 100 μl containing 1 × 10^6^ of either DAZL-P2A–GFP+ or GFP+ NT2 cells were injected subcutaneously into the left or right armpit separately. Teratomas were dissected after 8 weeks and tissues were processed and sectioned for hematoxylin and eosin (H&E) staining and RNA extraction.

### RNA extraction and RT–qPCR

Total RNA was extracted from cells using TRIzol™ Reagent (15596026, Thermo Fisher Scientific). For the quantification of mature miRNA expression, reverse transcription was performed using the miDETECT A Track miRNA qRT-PCR Starter Kit (C10712, Ribobio). Quantitative real-time polymerase chain reaction (PCR) was performed using miDETECT A Track miRNA qPCR Primer (R11071.3, Ribobio). For the quantification of mRNA expression, reverse transcription was performed using PrimeScript RT Reagent Kit with gDNA Eraser (RR047A, TaKaRa). Quantitative real-time PCR was performed using TransStart Top Green qPCR Supermix (AQ131, TransGen) on Bio-Rad CFX96.

### eCLIP-seq

eCLIP was performed as previously described (34) with minor modifications. Briefly, for every 1 × 10^7^ cells, DAZL–RNA interactions were stabilized under UV cross-linking (254 nm, 3 × 200 mJ/cm^2^), followed by lysis in 500 μl of chilled lysis buffer [50 mM Tris–HCl, pH 7.4, 100 mM NaCl, 1% Igepal CA-630, 0.1% sodium dodecylsulfate (SDS), 0.5% sodium deoxycholate] for 15 min at 4°C, sonication for 1 min 30 s (30 s on, 30 s off), DNA digestion by 2 μl of Turbo DNase and limited digestion by RNase I. Immunoprecipitation of the DAZL–RNA complex was performed with 5 μg of primary antibody (Sigma, F1804) using 60 μl of Dynabeads protein G (ThermoFisher) at 4°C overnight, and stringent washes as follows, high salt buffer twice (50 mM Tris–HCl, pH 7.4, 1 M NaCl, 1% Igepal CA-630, 0.1% SDS, 0.5% sodium deoxycholate, 1 mM EDTA), wash buffer once (20 mM Tris–HCl, pH 7.4, 10 mM MgCl_2_, 0.2% Tween-20) and 1× FastAP buffer once (10 mM Tris–HCl, pH 7.4, 5 mM MgCl_2_, 100 mM KCl, 0.02% Tween-20). Immunoprecipitated RNAs were treated by FastAP and PNK, tagged with barcoded 3′ RNA linker (X2A, /5phos/ AAGUAUANNNNNAGAUCGGAAGAGCGUCGUGUAG/3SpC3/; X2B, /5phos/AGAAGAUNNNNNAGAUCGGAAGAGCGUCGUGUAG/3SpC3/) by T4 RNA ligase in a high concentration of polyethylene glycol (PEG) 8000, electrophoresed on NUPAGE 4–12% Bis-Tris protein gels (ThermoFisher) and transferred onto a nitrocellulose membrane. A region of ∼100 kDa above DAZL were excised, treated with proteinase K and Trizol to isolate RNAs. Isolated input RNAs were further treated with FastAP and PNK, purified by MyONE Silane Dynabeads (ThermoFisher), ligated with RiL19 3′ RNA adaptor (/5Phos/AGAUCGGAAGAGCGUCGUG/3SpC3/) using T4 RNA ligase, purified by MyONE Silane Dynabeads and reverse transcribed with AffinityScript (Agilent). Before 5’ linker ligation, input RNAs were treated with ExoSAP-IT (Affymetrix) to remove excess oligonucleotides and purified by MyONE Silane Dynabeads. Ligation of the 5′ linker (/5Phos/ NNNNNNNNNNAGATCGGAAGAGCACACGTCTG/3SpC3/) was perfomed on beads by T4 RNA ligase with a high concentration of PEG8000 and dimethylslfoxide (DMSO). After cleanup with MyONE Silane Dynabeads, RNAs were quantified by qPCR, amplified by Q5 (NEB), purified by Ampure XP beads and size selected by electrophoresis. Samples were sequenced by Illumina HiSeq X Ten.

### Quantification and statistical analysis

Statistical details of analysis including statistical test used, value of *n* and statistical significance are all described in the figure legends.

### Scatterplot and Pearson correlation coefficient (PCC) analysis for IF co-localization

The cell fluorescence images were all taken by Nikon super-resolution laser confocal microscopy (A1R MP multiphoton, Nikon). Scatterplot analysis was conducted by using confocal microscope images, and fluorescence intensity was calculated with ImageJ software (35). Images were analyzed for co-localization with NIS Elements Imaging Software (Nikon) using the co-localization module to calculate the PCCs (36).

### miRNA-seq data processing

The reads were mapped to the human reference genome hg19 (downloaded from the UCSC genome browser) by mapper.pl script in mirdeep2 (37). miRNA mature and hairpin sequences were downloaded from miRBase. The miRNA read counts were obtained by mirdeep2. Then the count data were normalized across libraries using the trimmed mean of M-values (TMM) normalization method. The differentially expressed miRNAs were obtained by using edgeR (38), which uses a negative binominal distribution to capture the quadratic mean–variance relationship.

### miRNA-seq differential expression analysis

**Figure 1.**
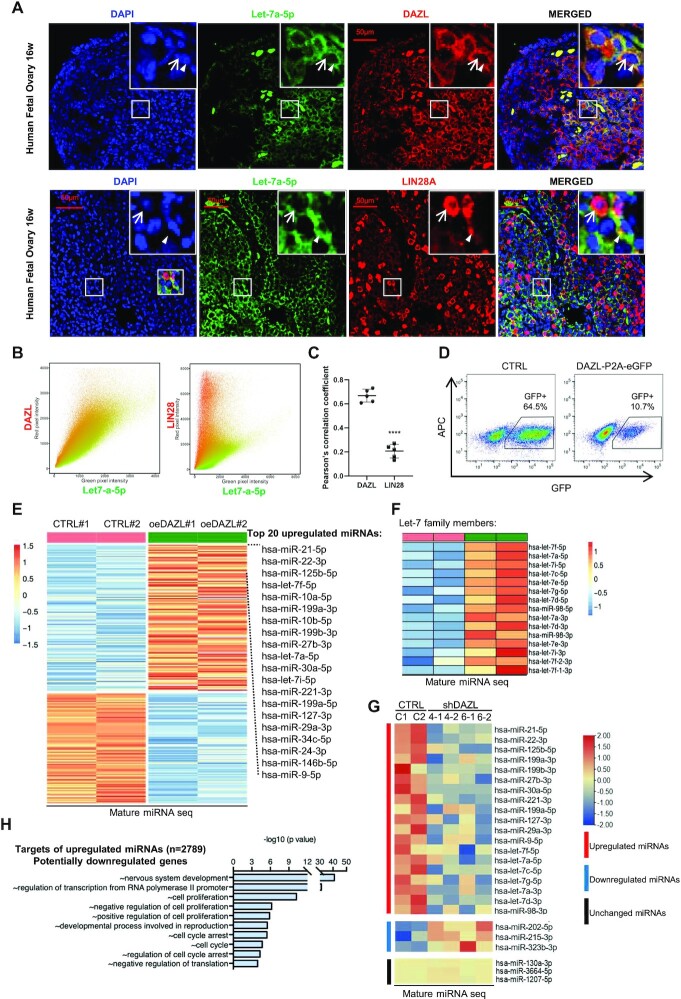
DAZL up-regulates mature miRNA levels in hPGCs. (**A**) FISH/immunofluorescence of let-7a-5p and DAZL, let-7a-5p and LIN28A of 16-week-old human fetal ovary. Co-staining of let-7a-5p FISH (green) and DAZL immunofluorescence (red) in human fetal ovary. The white arrow indicates DAZL- or LIN28A-positive cells and the arrowhead indicates let-7a-5p-positive cells. Scale bar, 50 μm. (**B**) Scatterplots of let-7a-5p staining intensity (green) versus DAZL or LIN28A staining intensity (red) in the merged images of (A). (**C**) PCC analysis of the co-localization of let-7a-5p-positive staining with DAZL or LIN28A stainings. Five merged regions were quantified; each contains ∼100 cells. (**D**) FACS analysis of cells overexpressing DAZL-P2A–eGFP or eGFP (CTRL). Gating represents the GFP+ population and its percentage. APC is an irrelevant channel for detecting the level of autofluorescence. (**E**) Differentially expressed mature miRNAs after DAZL overexpression (oeDAZL) compared with the control group (CTRL). # represents different biological repeats. (**F**) Let-7 family members among up-regulated miRNAs in (E). (**G**) Heatmap analysis of mature miRNA expression in control versus shDAZL hPGCLCs. The representative up-regulated, down-regulated and unchanged miRNAs were selected based on the results in (E). Duplicates of each group are shown. Two sets of shDAZLs (shDAZL4 and shDAZL6) were included. (**H**) Gene Ontology analysis of biological process enrichment of predicted targets of the DAZL up-regulated miRNAs.

Significantly up-regulated miRNAs were defined as miRNAs with a >1 log fold change (FC) with a false discovery rate (FDR) ≤0.15, and significantly down-regulated miRNAs were defined as miRNAs with a smaller than –1 logFC with an FDR ≤0.15. The miRNAs in Figure 1 were ranked in descending order by logCPM (counts per min) in the up-regulated or down-regulated group separately. Target sites were predicted using multiMiR (39) with ‘miranda’, ‘pictar’ and ‘targetscan’ selected as databases. Predicted target sites in this study were defined by combining the top 30 000 predictions from each database. The cut-off number was set at 30 000 in each database to avoid a dominating effect caused by databases with very large numbers of predictions. Entrez Gene Identifiers were converted to Gene Symbols using ‘org.Hs.eg.db’ (version 3.4.1).

### Quantification of precursor and mature miRNA expression levels

Cutadapt (v1.18) was used for trimming the adaptor (AAAAAAAAAA). Additionally, the first 3 nt of each read was trimmed. Subsequently, reads were filtered to retain only reads of lengths >15 nt. Adaptor-trimmed reads uniquely aligned to the miRBase with Bowtie (v0.12.7). After counting raw read coverage per pre- or mature miRNA annotation with the featureCounts(v2.0.1) package, reads were input into R version 4.0.1, and the R package DESeq2 was used to perform a differential expression analysis.

### RNA-seq data analysis

Raw sequence reads were initially processed using FastQC (v0.11.8) for quality control. Adaptor sequences and poor-quality reads were removed using TrimGalore (v0.6.1). Quality-filtered reads were then mapped to the human genome (hg38) using hisat2 (v2.1.0), and only the uniquely mapped reads were kept. Read counts were calculated using Stringtie (v2.1.4). Differentially expressed genes (DEGs) were identified using the R package DESeq2 (FC ≥2 and *P*-value ≤0.05).

### Bioinformatic analysis of eCLIP data

Adaptors were trimmed from original reads using Cutadapt (v1.18) with default setting. Next, PCR duplicates and random barcodes were removed using fastq2collapse.pl. Bwa (v0.7.17) was used to align reads to hg38. Only the uniquely mapped reads were retained. All reads remaining after a second run of PCR duplicate removal were regarded as usable reads.

## RESULTS

### DAZL up-regulates mature miRNA levels in hPGCs

To test whether there is a correlation between DAZL and miRNA in germ cells *in vivo*, we first examined the expression levels of both DAZL and let-7a-5p, which belong to the let-7 miRNA family, in a 16-week-old human fetal ovary. Double staining of DAZL and let-7a-5p showed that late PGCs, which are located in the inner ovary, expressed high levels of DAZL and also expressed high levels of let-7a-5p (Figure 1A). In contrast, PGCs which highly expressed LIN28A (40) showed low or no expression of let-7a-5p. A control staining section without the let-7a-5p probe showed a low number of non-specific stainings which were not cytoplasmic (Supplementary Figure S1A). Both scatterplot and PCC analysis (let-7a-5p + DAZL = 0.67, let-7a-5p + DAZL = 0.21) of the double stainings confirmed that the let-7a-5p-positive stainings and cells mostly co-localized with DAZL but not LIN28A (Figure 1B, C).

Therefore, we examined the effect of DAZL overexpression on miRNA, and measured the level of mature let-7 during *in vitro* differentiation of hPGCLCs. The hPGCLCs overexpressing human DAZL (oeDAZL) showed significantly higher levels of mature let-7a-5p from day 2 to day 6 than the control groups (Supplementary Figure S1B). To further confirm that increased levels of let-7a-5p were caused by oeDAZL, we isolated oeDAZL cells at day 6 via FACS and found that the oeDAZL group showed four times the amount of let-7a-5p than the control group (Figure 1D; Supplementary Figure S1C).

As oeDAZL may affect the levels of other miRNAs, we performed miRNA sequencing to comprehensively profile all the miRNAs potentially regulated by DAZL. The results showed that 207 miRNAs were differentially expressed between the control and oeDAZL hPGCLCs, of which 118 were up-regulated and 89 were down-regulated (Figure 1E). Based on their expression levels, the top 20 up-regulated miRNAs included several other miRNAs outside the let-7 family, such as miR-21-5p, miR-22-3p, miR-125b-5p, miR-10b-5p and miR199a-3p (Figure 1E). Let-7a-5p was not the only member of the let-7 family that was up-regulated in the oeDAZL cells. Eight out of nine members of the let-7 family were among the most significantly up-regulated miRNAs in oeDAZL cells, including let-7f-5p, let-7a-5p and let-7i-5p (Figure 1F). Some of these up-regulated miRNAs have been previously detected in mouse germ cells, including many let-7 family members (20,21,40), whereas several others were newly identified miRNAs that potentially play important roles in regulating late PGC development. To further validate the regulation of these miRNAs by DAZL, we silenced DAZL using shRNA in hPGCLCs and examined the expression levels of the miRNAs that were up-regulated in the oeDAZL group. Both of the shDAZLs were used in the previous study (32) and validated again in this study for their silencing effects (Supplementary Figure S1D). Comparing the shDAZL groups (duplicates of two shDAZL4 and shDAZL6) with the control cells, the average expression levels of the 118 up-regulated mature miRNAs by oeDAZL were down-regulated in the shDAZL hPGCLCs, and 93 of these miRNAs were significantly lower than the controls (Supplementary Figure S2A–C). In contrast, the average expression levels of the 89 down-regulated mature miRNAs were up-regulated by shDAZL, and 62 of these miRNAs were significantly lower than in the controls (Supplementary Figure S2D–F). Representatives of the up-regulated, down-regulated and unchanged miRNAs demonstrated the expected trends of expression upon shDAZLs (Figure 1G).

Next, we analyzed the cellular and molecular processes in which these 207 miRNAs potentially participated. The MultiMiR program was used to predict the targeted genes of these up-regulated miRNAs (39). Three independent miRNA databases (‘miranda’, ‘pictar’ and ‘targetscan’) predicted 2789 potentially down-regulated genes (Figure 1H). Notably, among the enriched genes involved in various processes, multiple Gene Ontology (GO) terms appeared to be related to ‘cell proliferation’ and ‘cell cycle’. To validate the predictions, we conducted RNA sequencing of the control and oeDAZL cells, followed by analyzing the expression trends of the predicted gene targets based on both the up-regulated and down-regulated miRNAs. According to the RNA-seq analysis, there were 459 up-regulated DEGs and 1319 down-regulated DEGs between the control and oeDAZL cells (Supplementary Figure S3A). On the other hand, among the predicted targets of the top 20 up-regulated miRNAs, the majority of genes (57%) are down-regulated based on the RNA-seq of the control and oeDAZL cells, and GO analysis of these genes showed that they were involved in ‘nervous system development’, ‘regulation of cell cycle’, ‘positive regulation of cell proliferation’ and other processes (Supplementary Figure S3B–D). In contrast, the majority of genes (49%) were up-regulated among the predicted targets of the top down-regulated miRNAs, and the GO analysis of these genes indicated that they were involved in ‘nervous system development’, ‘positive regulation of transcription from RNA polymerase II promoter’, ‘negative regulation of cell proliferation’ and other processes. Interestingly, the predicted targets of the up-regulated miRNAs included many genes involved in positive regulation of cell proliferation, whereas the predicted targets of the down-regulated miRNAs included negative regulation of cell proliferation, and both were validated by RNA-seq.

The analyses suggest that one of the major cellular processes regulated by DAZL during *in vitro* differentiation of PGCs and the *in vivo* transition from early to late PGCs is cell proliferation, and this process might be regulated by increasing miRNAs that target genes involved in cell proliferation or the cell cycle.

### DAZL co-localizes with DICER and co-immunoprecipitates with precursor miRNA

**Figure 2.**
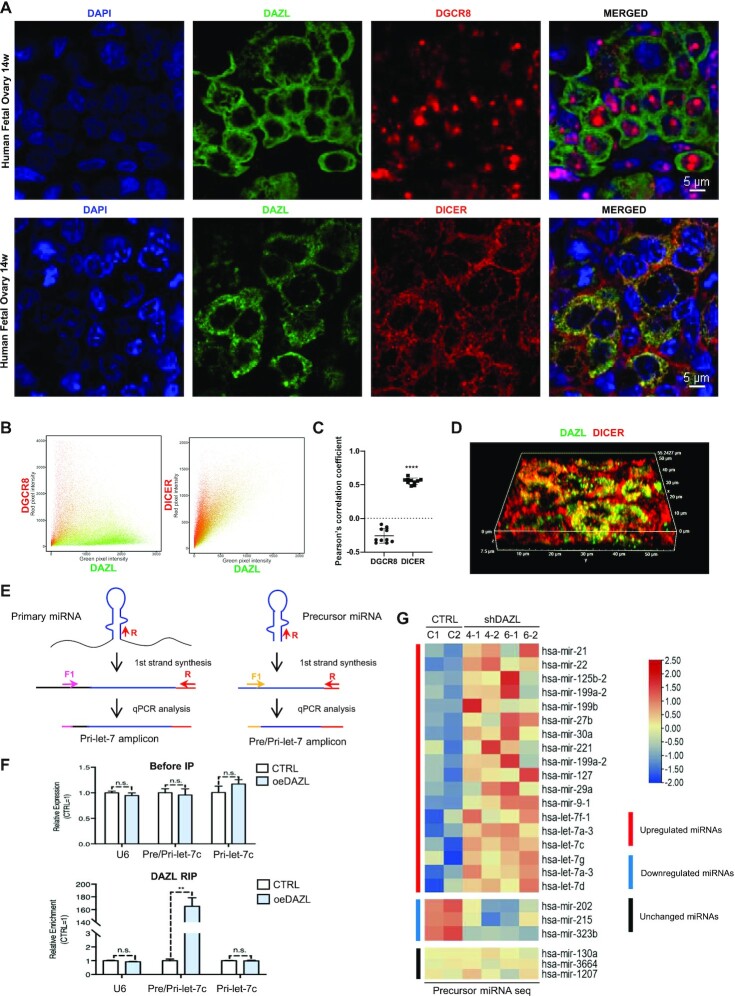
DAZL co-localizes with DICER and co-immunoprecipitates with pre-miRNA. (**A**) Immunostaining of DAZL and DGCR8, and DAZL and DICER of 14-week-old human fetal ovary. Scale bar, 5 μm. (**B**) Scatterplots of DAZL (green) versus DGCR8 or DICER staining intensity (red) in the merged images of (A). (**C**) PCC analysis of the co-localization of DAZL-positive staining with DGCR8 or DICER staining. Ten merged regions were quantified, and each contains >30 cells. (**D**) High resolution 3D imaging of DAZL and DICER immunostaining of 12-week-old human fetal ovary. Scale bar, 5 μm. (**E**) Schematic diagram showing qPCR analysis of primary and precursor/primary miRNAs. The pink arrow represents the forward primer of primary miRNA, the yellow arrow represents the forward primer of pri-/pre-miRNA, the red arrow represents the reverse primer of primary and pre-miRNA. (**F**) Relative expression of U6, pri-/precursor and precursor let-7c in the V5-DAZL group (oeDAZL) and DAZL group (CTRL) before and after RIP. Data shown are the means ± standard deviation (SD), *n* = 3. n.s., no significance. **P <0.01 (Student's *t*-test, two-sided/unpaired). (**G**) Heatmap analysis of pre-miRNA expression in CTRL versus shDAZL hPGCLCs. Two sets of shDAZL (shDAZL4 and shDAZL6) were included. The representative up-regulated, down-regulated and unchanged miRNAs were selected based on the results in Figure 1E. Duplicates of each group are shown.

According to the reported canonical biogenesis of miRNAs (41–44), there are multiple steps in which DAZL may participate in up-regulating the mature miRNAs, including transcriptional activation, pri-miRNA processing and pre-miRNA processing. DAZL has no reported transcriptional activity; therefore, it is more likely to participate in the processing steps for increasing mature miRNAs. The two processing steps are governed by two different protein complexes at distinct subcellular locations. Primary miRNAs are processed by DROSHA–DGCR8 complexes to generate pre-miRNAs in the nucleus, whereas pre-miRNAs are processed by DICER complexes to produce mature miRNAs in the cytoplasm (22,23). Thus, the subcellular localization of DAZL with either complex in hPGCs suggests the step in which DAZL may participate. Immunostaining of DAZL and DGCR8 in the fetal ovary hPGCs indicated that DAZL was expressed predominantly in the cytoplasm, whereas DGCR8 localized almost exclusively in the nucleus (Figure 2A; Supplementary Figure S4A). In contrast, independent immunostaining experiments showed the co-localization of DAZL with DICER in the cytoplasm of many hPGCs. Scatterplot and PCC analysis verified the co-localization of DAZL and DICER (Figure 2B, C). High-resolution 3D imaging indicated that many strong DAZL granule-like stainings co-localized with DICER at the same 3D positions in hPGCs (Figure 2D; Supplementary Movie S1). These results suggest that DAZL is more likely to function in the second step of miRNA processing, which occurs in the cytoplasm.

Next, through RIP, we tested the possibility that DAZL promotes the processing of pri- or pre-miRNAs to mature miRNAs. snRNA U6 was used as a negative control, and the levels of pri-let-7c and pre/-pri-let-7c were measured before and after RIP. The results showed that the levels of snRNA U6, precursor and primary let-7c were similar between the oeDAZL (control) and oeDAZL-V5 cell lysates prior to RIP (Figure 2E, F), but the level of pre-/pri-let-7c was significantly higher in the oeDAZL-V5 groups and higher than the pri-let-7c following RIP (Figure 2F). After quantifying and normalizing the pre/pri-let-7c, pri-let-7c for the adjusted pre-miRNA quantifications, precursors of let7c, miR199a and miR10a, but not primary miRNAs, were found to be enriched in the DAZL-V5 RIP fractions (Supplementary Figure S4B). Taken together, the above results suggest that DAZL directly bound to the precursors of the up-regulated miRNAs in the second step of miRNA processing in the cytoplasm of hPGCs.

To determine whether DAZL exhibited binding preferences to the pre-miRNAs of the up-regulated and down-regulated miRNAs in hPGCLCs, we performed eCLIP-seq. The results showed that DAZL bound to 10 out of 10 pre-/pri-miRNAs of the up-regulated miRNAs, but none of the top 10 down-regulated miRNAs (Supplementary Figure S5). Although the eClip-seq results did not discern the binding of DAZL to pre- or pri-miRNAs of the up-regulated miRNAs, no binding to the down-regulated miRNA site excluded the possibility of any binding of DAZL to these pre- or pri-miRNAs. Moreover, our RIP results utilizing qPCR to quantify DAZL binding (Figure 2E, F; Supplementary Figure S4B) showed much higher binding of DAZL to pre-miRNAs than pri-miRNAs. Taken together, we concluded that DAZL preferentially bound to pre-miRNAs of the up-regulated miRNAs but not pre-miRNAs of the down-regulated miRNAs.

In contrast to mature miRNAs, pre-miRNAs of the up-regulated miRNAs were expected to decrease upon DAZL silencing. We validated the expression trends of the pre-miRNAs using shDAZLs as in Figure 1G and confirmed the opposites trend of the expression of the pre-miRNAs (Figure 2G).

### DAZL directly binds pre-let-7 and specifically recognizes the GUU motif in the loop

**Figure 3.**
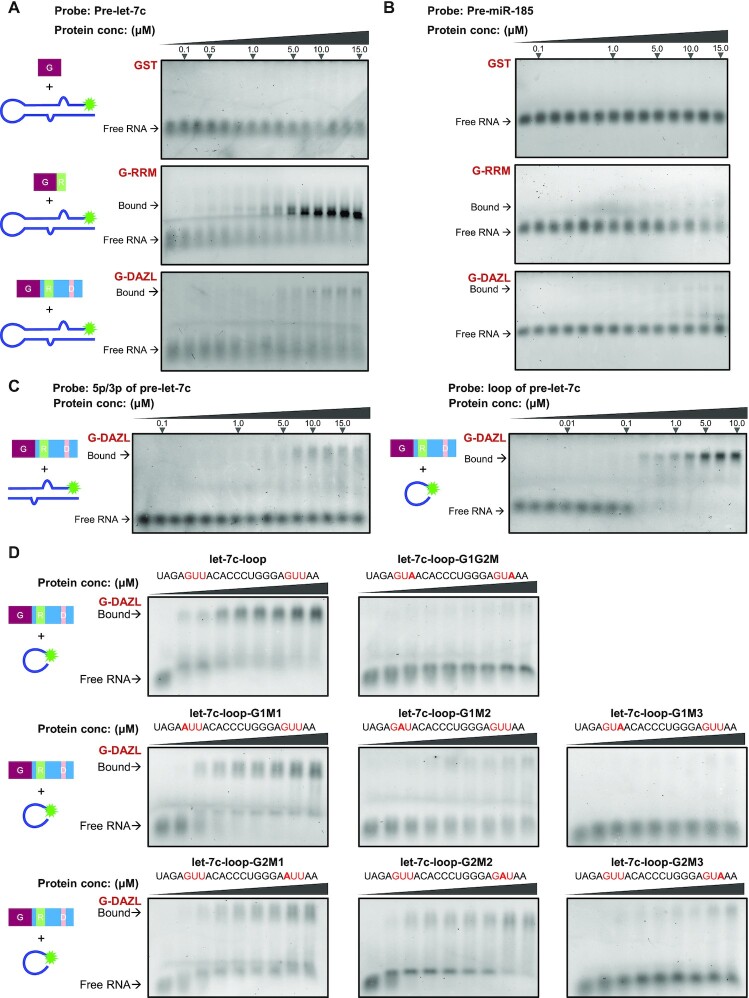
DAZL directly binds pre-let-7 and specifically recognizes the GUU motif in the loop. (**A**) EMSA of direct binding of purified GST, GST–DAZL and GST–RRM to FAM-labeled pre-let-7c probe. The purple rectangle represents GST, the green rectangle represents the RRM domain of DAZL, the pink rectangle represents the DAZ repeat domain of DAZL, the blue rectangle represents the other region of DAZL except from the RRM and DAZ repeat, and the green asterisk represents FAM fluorescence labeling at the N-terminus of the miRNA probe. (**B**) EMSA of direct binding of purified GST, GST–DAZL and GST–RRM to FAM-labeled pre-miR-185 probe. (**C**) EMSA of direct binding of purified GST–DAZL to the loop and mature sequence of pre-let-7c. 5p/3p of the pre-let-7c probe represents the mature sequence of pre-let-7c with FAM fluorescence labeling at the N-terminus of the 5p strand. The loop of the pre-let-7c probe represents the loop region of pre-let-7c with FAM fluorescence labeling at the N-terminus of the loop. (**D**) EMSA of direct binding of purified GST–DAZL to the loop sequence of pre-let-7c with a mutation within the GUU motif. The let-7c-loop contains two GUU motifs (light red). The first G, the second U and the third U of each GUU motif were mutated into A (dark red).

To determine whether DAZL can bind pre-miRNAs directly *in vitro*, we purified recombinant DAZL (Supplementary Figure S6A) and conducted EMSA. Because the RRM of DAZL alone was reported to bind specific RNA sequences (16), we also purified the DAZL RRM domain as a positive control (Supplementary Figure S6A). EMSA was performed using the purified proteins and refolded pre-let-7c probes with fluorescence-labeled FAM (Figure 3A; Supplementary Figure S6B). The addition of GST–DAZL at ∼1.0 μM, but not the negative GST control, resulted in a shifted band of pre-let-7c probes (Figure 3A). Moreover, GST–RRM showed a stronger binding ability to pre-let-7c than GST–DAZL. These results indicated that DAZL and its RRM can directly bind to the precursor let-7 *in vitro* and the RRM has strong affinity for the pre-miRNA. To confirm the preferential binding of the DAZL RRM on pre-miRNAs, we performed EMSA using the RRM with the other two up-regulated miRNAs, pre-miR199a and pre-let-7i, as well as a not significantly up-regulated miRNA, pre-miR-185. The results showed that DAZL can strongly bind to pre-let-7c, pre-let-7i and pre-miR-199a, but not pre-miR-185 (Figure 3B; Supplementary Figure S7A–C), indicating that DAZL, especially the RRM domain of DAZL, can directly and preferentially bind to specific pre-miRNAs.

The pre-let-7 consists of the stem region and loop region (45). To determine the pre-let-7-binding site of DAZL, we divided the pre-let-7 into stem double-stranded RNA (dsRNA) and loop single-stranded RNA (ssRNA) probes, named pre-let-7-3p/5p and pre-let-7-loop, respectively. EMSA and FP results demonstrated that DAZL more favorably bound to the pre-let-7 loop than pre-let-7-3p/5p (Figure 3C; Supplementary Figure S7D, E).

To examine whether the GUU motif may dictate the binding preferences, we first analyzed the precursor sequences of the 118 up-regulated and 89 down-regulated miRNAs using a program recently applied to find motifs at the mRNA splicing sites (46). Although there appeared to be more GUU motifs (showing as GTT in the logo graph) in the precursor sequence of the up-regulated than of the down-regulated pre-miRNAs, there was no definite GUU motif at a specific position based on the results of the motif search program (Supplementary Figure S8A).

Because the DAZL RRM specifically recognizes a GUU triplet and substitutions in the GUU triplet decrease the affinity of the RRM (16), we next compared and analyzed the sequence of the pre-let-7-3p/5p and pre-let-7-loop. Both the loop and stem regions of pre-let-7 contained two separate GUU motifs (Supplementary Figure S6B). DAZL preferentially bound to the loop regions (Figure 3C), so we performed a single base mutation for each of the two GUU motifs in the pre-let-7c loop to test whether the binding of DAZL to loops was GUU dependent. The results showed that mutation of the third U to A in both GUUs significantly disrupted the binding of DAZL to the loop, whereas mutations in the first G and the second U to A only slightly affected binding (Figure 3D). Moreover, the mutation of the base next to GUU to U did not significantly affect the binding ability of DAZL to the loop (Supplementary Figure S8B). The above results indicate that DAZL bound to the loop through recognition of the GUU motif. Moreover, the third U in GUU was essential in the binding of DAZL, which is consistent with the reported binding specificity of RRM on the 3′-UTR of mRNA.

### DAZL interacts with and enhances DICER activity *in vitro*

**Figure 4.**
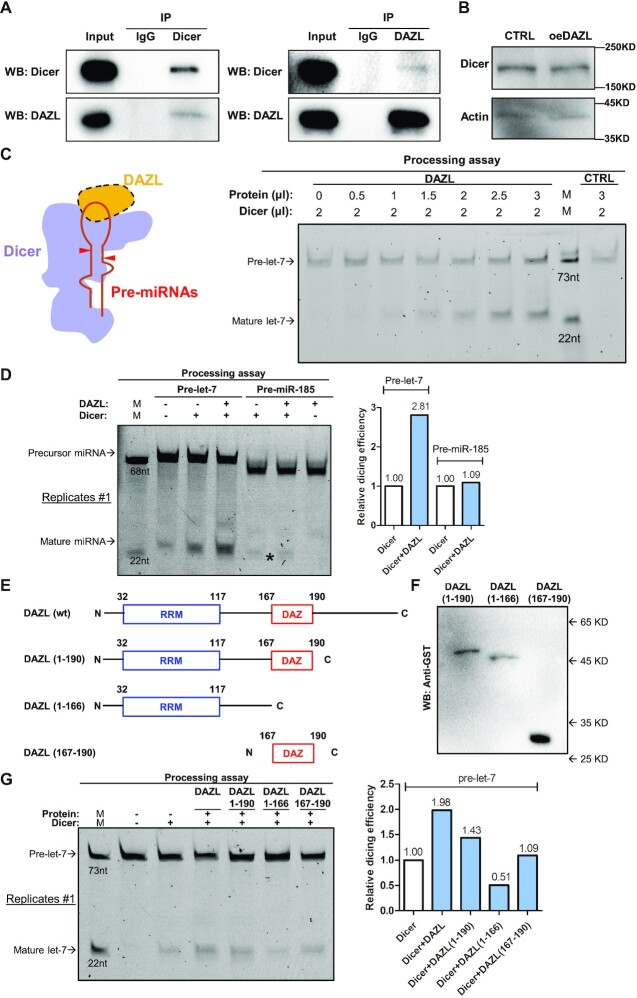
DAZL interacts with and enhances DICER activity *in vitro*. (**A**) Co-IP of endogenous DICER and overexpressed DAZL in 293FT cells. DAZL was overexpressed by plasmid transfection in 293FT cells. Whole-cell lysates were used for co-IP. RNA has been removed by an RNase A/T1 cocktail prior to co-IP. Normal rabbit IgG was used as a negative control. (**B**) Western blot of DICER in the DAZL-overexpressing group (oeDAZL) and control group (CTRL) in 293FT cells. (**C**) DAZL dose–response analysis of pre-let-7 cleavage by DICER in the presence of different concentrations of purified GST–DAZL proteins. A hypothetical model of pre-let-7 cleavage by DAZL and DICER (left). Yellow represents DAZL, the red hairpin represents pre-let-7, the red triangle represents DICER cleavage sites and purple represents DICER. In the processing assay (right), DAZL and 2 μl of 100 nM DICER were added to 100 pM FAM-labeled pre-let-7 and the reaction was incubated for 15 min at 37°C before running 8 M urea polyacrylamide gel electrophoresis (PAGE). CTRL represents GST proteins. (**D**) Pre-let-7 and pre-miR-185 cleavage by DICER in the presence of the same concentration of purified GST–DAZL proteins. Proteins were added to 100 pM FAM-labeled pre-let-7 and the reaction was incubated for 15 min at 37°C before running 8 M urea PAGE. In the grayscale statistics histogram (right), the relative dicing efficiency of each group (blue column) was normalized to the control group (white column), and the number above each column represent the difference in gray levels compared with the control group. * denotes the faint bands of mature miR-185. (**E**) Illustration depicting the truncated DAZL proteins. The numbers in parentheses represent the amino acid position of this protein corresponding to DAZL. (**F**) Western blot of recombinant GST-tagged truncated DAZL proteins from *E. coli*. Anti-GST antibodies were used. (**G**) Pre-let-7 cleavage by DICER in the presence of the same concentrations of purified GST-tagged DAZL, DAZL (1–190), DAZL (1–166) and DAZL (167–190) proteins. Truncated DAZL proteins were added to 100 pM FAM-labeled pre-let-7 and the reaction was incubated for 15 min at 37°C before running 8 M urea PAGE. In the grayscale statistics histogram (right), the relative dicing efficiency of each group (blue column) was normalized to the control group (white column), and the number above each column represents the difference in gray levels compared with the control group.

As the precursor miRNA loop position is crucial in DICER recognition, for ensuring precise processing (27,47), we wondered whether DAZL can interact with the DICER complex and participate in dicing activity. We performed protein co-IP with DICER antibodies or DAZL antibodies in DAZL-overexpressing 293FT cells after an RNase treatment. As expected, we detected both DAZL in the IP products of DICER and DICER in the IP products of DAZL (Figure 4A), while independent western analyses ensured that the overexpression of DAZL did not affect the expression level of DICER (Figure 4B). These results indicate that DAZL can interact with DICER and this interaction is not RNA dependent.

There are several DICER- and RNA-binding proteins, such as TRBP and PACT, that can influence the DICER activity (25,29). The interactions of DAZL and DICER led us to ask whether DAZL may also influence DICER activity in miRNA processing. To test this possibility, we performed *in vitro* processing of pre-let-7 in the presence of full-length DAZL with the purified DICER published recently (48). The addition of the recombinant DAZL protein significantly enhanced the cleavage of pre-let-7 by DICER compared with the control, and the cleavage level was DAZL dose dependent and reproducible (Figure 4C; Supplementary Figure S9A, B). We then evaluated the ability of DAZL to facilitate the cleavage of pre-let-7 versus pre-miR-185 to explore whether the differential binding ability of DAZL to pre-miRNAs may affect miRNA cleavage. The results showed that DAZL enhanced dicing efficiency on pre-let-7 but not pre-miR-185 (Figure 4D; Supplementary Figure S9C). Taken together with the direct binding of DAZL to the miRNA loop described above, the results indicate that DAZL proteins can directly bind to the loop region of precursor let-7 and enhance the processing from pre-let-7 to mature let-7 with DICER *in vitro*.

Next, we aimed to determine the domain of DAZL that is most important in facilitating pre-miRNA cleavage. Therefore, we expressed and purified DAZL proteins containing different protein domains (Figure 4E, F, Supplementary Figure S10A). We examined the effect of these proteins on DICER-mediated *in vitro* cleavage of pre-let-7 and found that full-length DAZL had the strongest enhancing effect, DAZL (1–190) was the second strongest, DAZL (167–190) had no significant enhancing effect and DAZL (1–166) inhibited cleavage (Figure 4G; Supplementary Figure S10B). We speculated that the inhibition of cleavage by DAZL (1–166) may have been caused by a lack of interacting domains with DICER, despite its strong ability to bind to miRNAs. In summary, the full-length version of DAZL is necessary to ensure efficient miRNA cleavage, and the specific binding of DAZL to miRNAs affects the cleavage of miRNAs, ultimately promoting the formation of different mature miRNAs.

### DAZL-up-regulated miRNAs inhibit proliferation and the cell cycle of PGCLCs

**Figure 5.**
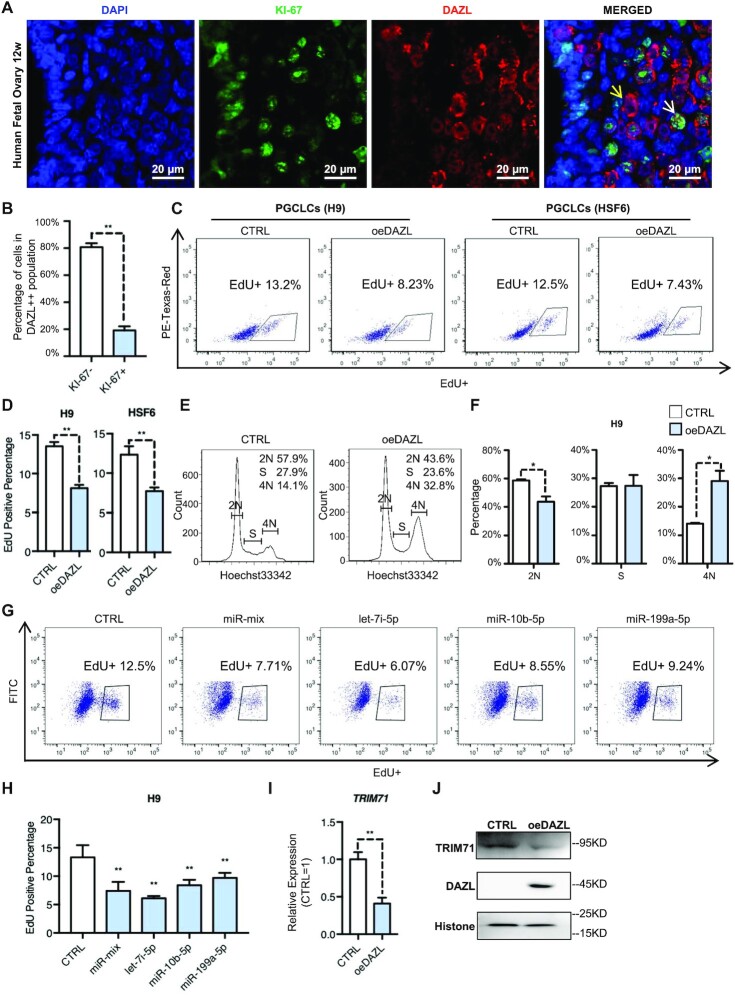
DAZL-up-regulated miRNAs inhibit proliferation and the cell cycle of PGCLCs. (**A**) Immunostaining of DAZL and KI-67 of 12-week old human fetal ovary. The yellow arrow represents KI-67-negative cells. White arrow represents KI-67-positive cells. Scale bar, 20 μm. (**B**) Percentage of the KI-67 signal in DAZL++ cells in (A). Data show are the mean ± SD, *n* = 5 independent areas. **P <0.01 (Student's *t*-test, two-sided/unpaired). (**C**) FACS analysis of EdU incorporation of the DAZL-overexpressing group (oeDAZL) and control group (CTRL) in PGCLCs (H9, left) and PGCLCs (HSF6, right) on day 6. Gating represents the EdU+ population and its percentage. PE-Texas-Red is an irrelevant channel for detecting the level of autofluorescence. (**D**) Quantification of the EdU-positive percentage in (C). Data show the mean ± SD, *n* = 3. **P <0.01 (Student's *t*-test, two-sided/unpaired). (**E**) Cell cycle analysis of the oeDAZL and control group in PGCLCs (H9) on day 6. The numbers in the upper right corner represent the percentage of each group. (**F**) Quantification of the 2N, S and 4N cell percentage in (E). Data show are the mean ± SD, *n* = 3. **P <0.01, *P <0.05 (Student's *t*-test, two-sided/unpaired). (**G**) FACS analysis of EdU incorporation of a single miRNA-overexpressing group (let-7i-5p, miR-10b-5p and miR-199a-5p), a mixed miRNA-overexpressing group (miR-mix) and the control group in H9 on day 6. Gating represents the EdU+ population and its percentage. FITC is a reference channel for detecting the level of autofluorescence. (**H**) Quantification of the EdU-positive percentage in (G). Data show the mean ± SD, *n* = 4. **P <0.01 (one-way ANOVA followed by multiple comparisons with control). (**I**) Relative expression of *TRIM71* in the oeDAZL and control group in PGCLCs (H9) on day 6. Normalized to GAPDH, CTRL = 1. Data show the mean ± SD, *n* = 9. **P <0.01 (Student's *t*-test, two-sided/unpaired). (**J**) Western blot of TRIM71 in the oeDAZL and control group in PGCLCs (H9) on day 6.

Because the GO analysis of the predicted targets showed that the DAZL-up-regulated miRNAs may regulate cell proliferation (Figure 1H), we first examined the correlation between DAZL and PGC proliferation in human fetal ovaries by examining a marker of active proliferating cells, KI-67. Positive signals of KI-67 were largely detected in cells with undetected or low levels of DAZL expression, but the KI-67 level significantly decreased in germ cells with high levels of DAZL (DAZL++) (Figure 5A, B). Next, we evaluated cell proliferation following DAZL overexpression during *in vitro* PGC induction in two independent hESC lines, H9 and HSF6. The proliferation of cells labeled with EdU decreased significantly in the oeDAZL groups (average of 8.23% in H9, 7.43% in HSF6) compared with the control groups (average of 13.2% in H9, 12.5% in HSF6) in both cell lines (Figure 5C, D). In addition, we observed a significant decrease in the 2N population and an accumulation of 4N cells in the oeDAZL group (Figure 5E, F). These results confirmed the GO analysis results from the predicted targets of DAZL-regulated miRNAs.

Given that some miRNAs up-regulated by oeDAZL were expected to target genes involved in regulating cell proliferation, we sought to test whether the overexpression of these miRNAs without oeDAZL could down-regulate cell proliferation. We selected three miRNAs that were significantly up-regulated by DAZL. Overexpression of let-7i-5p, miR-10b-5p or miR-199a-5p reduced EdU-positive cells from ∼12.5% to 6.1, 8.6 and 9.2%, respectively (Figure 5G). Moreover, overexpression of all three miRNAs reduced the EdU-positive percentage to 7.7%. The EdU assay revealed that the overexpression of let-7i-5p, miR-10b-5p or miR-199a-5p sufficiently inhibited cell proliferation, and that let-7i showed the strongest inhibitory influence (Figure 5G, H).

On the other hand, it is expected that the targeted gene regulated by the miRNAs may be down-regulated in the oeDAZL cells. Indeed, the transcriptional and translational levels of *TRIM71*, a cell proliferation-related gene that is also a predicted target of the let-7 family (49–51) (Supplementary Figure S11A), was significantly down-regulated in the oeDAZL group to more than half of the level compared with the control cells (Figure 5I, J). To further confirm that the down-regulation of TRIM71 was due to the expression level of DAZL, we tested the effect of silencing DAZL on the *TRIM71* level. *TRIM71* expression was moderately increased when DAZL was knocked down (Supplementary Figure S11B). Therefore, the results support the notion that oeDAZL down-regulated cell proliferation by up-regulating miRNAs such as let-7.

### DAZL suppresses proliferation and tumorigenesis of N-tera2

**Figure 6.**
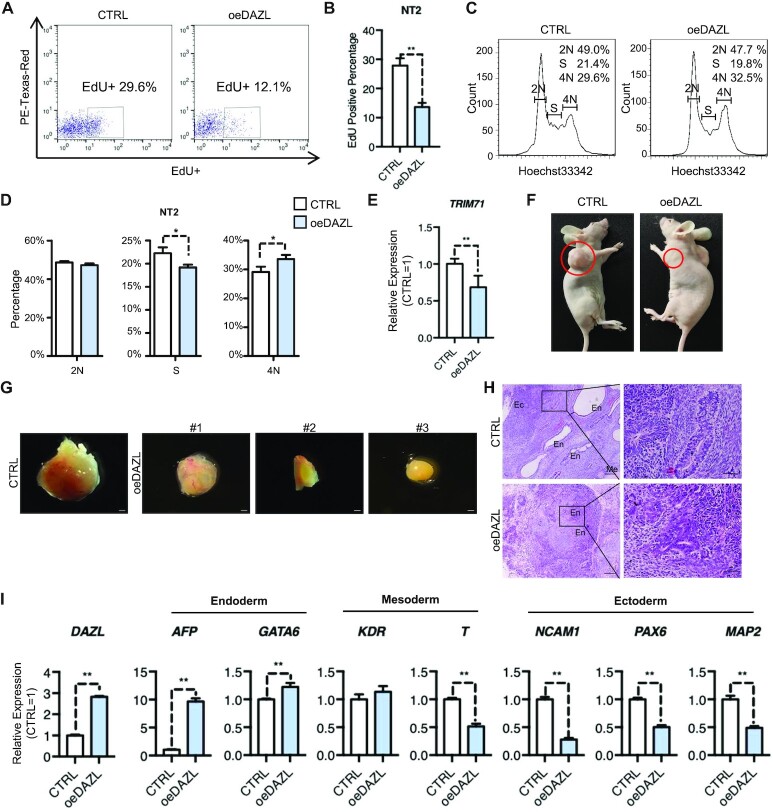
DAZL suppresses proliferation and tumorigenesis of N-tera2. (**A**) FACS analysis of EdU incorporation of the DAZL-overexpressing group (oeDAZL) and control group (CTRL) in NT2 cells. Gating represents the EdU+ population and its percentage. PE-Texas-Red is an irrelevant channel for detecting the level of autofluorescence. (**B**) Quantification of the EdU-positive percentage in (A). Data show the mean ± SD, *n* = 3. **P <0.01 (Student's *t*-test, two-sided/unpaired). (**C**) Cell cycle analysis of the oeDAZL and CTRL group in NT2 cells. The numbers in the upper right corner represent the percentage of each group. (**D**) Quantification of 2N, S and 4N cell percentage in (C). Data show the mean ± SD, *n* = 3. *P <0.05 (Student's *t*-test, two-sided/unpaired). (**E**) Relative expression of *TRIM71* in the oeDAZL and CTRL group in NT2. Normalized to GAPDH, CTRL = 1. Data show the mean ± SD, *n* = 9. **P <0.01 (Student's *t*-test, two-sided/unpaired). (**F**) Teratoma formation on the left and right armpits 8 weeks after injection of NT2 cells overexpressing DAZL (oeDAZL) or control (CTRL). The red circle represents the teratoma area. DAZL was overexpressed by virus infection in NT2 cells and isolated by FACS before injection. (**G**) Images of teratomas of the oeDAZL and CTRL group in NT2 cells. #1, #2 and #3 represent teratomas in independent mice. Scale bars, 1.14 mm. (**H**) Images of H&E staining in (G). En, endoderm; Me, mesoderm; Ec, ectoderm. Scale bars, 200 μm (left) and 50 μm (right). The magnified H&E results on the right are from the black box on the left. (**I**) Relative expression levels of three germ layer markers in teratomas. Data show the mean ± SD, *n* = 3. Normalized to GAPDH, CTRL = 1. **P <0.01 (Student's *t*-test, two-sided/unpaired). Endoderm markers, *AFP* and *GATA6*; mesoderm markers, *KDR* and *T*; ectoderm markers, *NCAM1*, *PAX6* and *MAP2*.

To determine whether the inhibitory effect of DAZL on cell proliferation also occurs in other germ cell types, we overexpressed DAZL in a human testicular germline tumor cell line, NT2. Accordingly, the overexpression of DAZL decreased EdU-positive populations of NT2 cells from ∼29.6% to ∼12.1%, and increased the ratio of 4N cells while decreasing the number of cells in S phase (Figure 6A–D). Similarly, oeDAZL NT2 cells showed lower expression levels of *TRIM71* (Figure 6E). To further confirm that the lower proliferation of NT2 after oeDAZL was due to an increase of the up-regulated miRNAs, we directly tested whether overexpression of let-7i-5p, miR-10b-5p and miR-199a-5p in NT2 cells may also decrease the proliferation. The results showed that a mixture of all three miRNAs significantly down-regulates the proliferation but individual miRNAs showed minor or no significant down-regulation of NT2 proliferation (Supplementary Figure S12), indicating the additive effects of the multiple miRNAs on cell proliferation of NT2.

We further examined the long-term effect of overexpressing DAZL in NT2 cells by generating teratomas in nude mice with control NT2 and oeDAZL NT2 cells. One million oeDAZL NT2 cells and control NT2 cells were implanted subcutaneously into the left and right arm of the same nude mice. Teratomas were observed on both sides after 8 weeks of implantation, but the teratomas formed via DAZL-overexpressing NT2 cells were much smaller than those formed by the control cells (Figure 6F, G). All three mice subjected to the same treatment showed differences in teratoma size between the oeDAZL and control cells (Figure 6G). The teratomas were examined histologically to determine whether NT2 cells could differentiate into different cell lineages. The results showed that control NT2 cell-derived teratomas contained tissues of all three germ layers, whereas oeDAZL NT2 cell-derived teratomas consisted mainly of tissues resembling endodermal morphology (Figure 6H). When the teratomas were examined for transcriptional expression, some markers of ectodermal and mesodermal lineages significantly decreased, whereas the markers of the endodermal lineage significantly increased in oeDAZL compared with the control teratomas (Figure 6I). This was consistent with the histological analysis. Collectively, these findings demonstrated that DAZL limited cell proliferation in both PGCs and NT2 cells. Moreover, the overexpression of DAZL in NT2 cells clearly suppressed teratoma growth.

## DISCUSSION

In this study, we elucidated that DAZL directly participated in miRNA biogenesis and regulated PGC proliferation. DAZL bound directly to the loop region of pre-miRNAs by recognizing the GUU motif and interacted with DICER to enhance DICER-mediated pre-miRNA cleavage. Furthermore, DAZL inhibited PGC proliferation through the up-regulated miRNAs. DAZL also inhibited the proliferation of germ cell-derived cancer cells (N-tera2) as well as impeding the development of teratomas.

DAZL recognized the GUU motif in the precursor of miRNAs, and changing the third U to A significantly reduced the binding of DAZL to the miRNAs. This specificity explains the differential up-regulation of a subset of miRNAs when DAZL was overexpressed in PGCLCs. For instances, the stem–loop regions of pre-let-7c, pre-let-7i and pre-miR-199a contain more than three GUU motifs, whereas pre-miR-185 only contains one. This specificity is consistent with previous findings that the RRM domain of DAZL can recognize GUU triplets of coding genes in their 3′-UTR (16). We further demonstrated that DAZL prefers to bind to the single-strand loop region of pre-miRNAs compared with the double-stranded stem region. The loop position of pre-miRNAs is critical in ensuring the accuracy of DICER processing (47). Thus, the binding of DAZL at the loop of pre-miRNAs may influence the recognition of DICER and pre-miRNA loading, resulting in isomiR production. Although isomiRs share the same seed sequence as miRNAs, they exhibit distinct functions (52–54). Accordingly, during DICER-dependent processing, DAZL may induce the production of alternative miRNA products, which suggests that DAZL may assist in miRNA length determination. Therefore, in germ cells, DAZL probably affects isomiR formation and influences the expression of different targeted genes. Recent studies reported that DICER processing activity of pre-miRNA is regulated by interactions of TRBP with LGP2 during viral infection (55–57), showing more examples of regulatory mechanisms of DICER activity by alternative protein interactions and structural characteristics of miRNAs.

A total of 118 miRNAs were identified as up-regulated miRNAs whereas 89 miRNAs were determined to be down-egulated miRNAs upon oeDAZL. The results of RIP, EMSA and eCLIP indicated that DAZL preferentially bound to the pre-miRNAs of the up-regulated miRNAs but not the down-regulated miRNAs. Hence, the effect of the up-regulation was a direct regulation of the up-regulated miRNAs but the effect of the down-regulation might be exerted through indirect regulation on the down-regulated miRNAs. For instance, DAZL might down-regulate the post-transcriptional expression of a transcription factor which was a transcriptional activator of the down-regulated miRNAs through 3′-UTR regulation. Suppression of transcription factors by DAZL through the 3′-UTR has been reported (58), so future study using transcriptome and translatome analysis (59) may help to identify the genes regulated by DAZL through post-transcriptional expression and validate the indirect down-regulation of the miRNAs.

**Figure 7.**
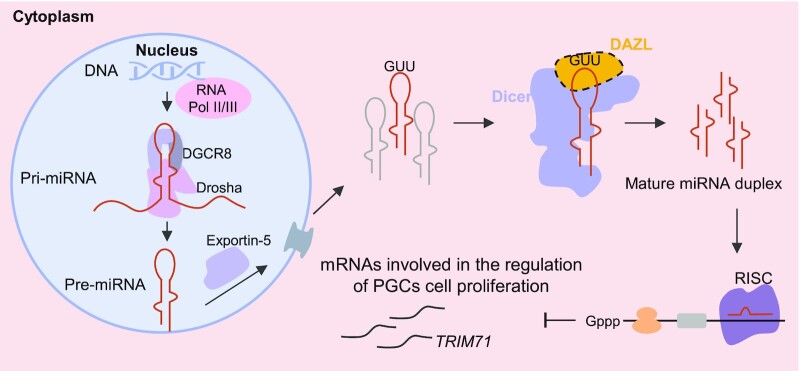
A model for DAZL binding directly to pre-miRNAs and enhancing miRNA biogenesis. During PGC development, DAZL (yellow) in the cytoplasm can bind directly to the loop region of pre-miRNAs (red hairpin structure) through recognizing the GUU motif. DAZL can also interact with DICER (blue) and promote DICER-mediated pre-miRNA processing to generate more mature miRNAs. Specific binding of DAZL to miRNAs affects the efficiency of DAZL in facilitating the cleavage of miRNAs. These miRNAs specifically promoted by DAZL are mainly involved in cell proliferation and cell cycle regulation.

The most obvious cellular processes we uncovered from GO analysis of the miRNA-targeted genes is cell proliferation. Moreover, germ cells with high expression of DAZL in human fetal ovary show significantly lower KI-67 staining, and a series of *in vitro* experiments in this study showed that DAZL overexpression is capable of down-regulating cell proliferation. Taken together, these data indicate that DAZL limits the proliferation of late PGCs by increasing some mature miRNAs such as the let-7 family and decreasing expression of genes positively regulating cell proliferation, including TRIM71 (Figure 7). The ability of DAZL to limit cell proliferation not only occurs in the *in vitro* differentiated PGCs, but also happens in NT2 testicular germline cells. Transplantation of NT2 cells into immunodeficient mice confirms that the suppression of cell proliferation persisted and the remaining oeDAZL NT2 cells are more likely to be of endodermal lineage. DAZL was reported to limit pluripotency of PGCs in both mouse and human PGCs (1,11,58), but regulation of proliferation has not been reported. If DAZL plays a role in regulating proliferation and pluripotency of human PGCs, it is more likely to participate in suppressing tumor formation. Interestingly, one recent study identified a variant near the DAZL gene in one patient with a testicular germ cell tumor (TGCT) (60), suggesting that a mutation causing abnormal expression of DAZL may lead to TGCT. Another example of a PGC gene associated with TGCT is DND1. Deletion mutation of Dnd1 in mice is known to cause a higher incidence of TGCT, and a heterozygous variant within a functional domain of DND1 was identified in a patient with TGCT, suggesting DND1 function to suppress TGCT in human germ cells (61).

In summary, this report identifies germ cell-specific DAZL as a novel enhancer of miRNA processing and reveals a new regulatory role for DAZL in PGC proliferation. Hence, DAZL has the ability to change the expression level of a gene through non-coding miRNAs beside its known function as a post-transcriptional regulator at the 3′-UTR of targeted genes. Moreover, other cellular processes may be regulated by DAZL through this mechanism because the targeted genes of miRNAs are not limited to the category of cell proliferation. Considering the proliferation inhibition and teratoma suppression capacity of DAZL, our findings may be useful for developing therapeutic treatments for patients suffering from germ cell tumors.

## DATA AVAILABILITY

All data shown are freely available. The sequencing datasets have been deposited in the GEO with accession number GSE206466.

## Supplementary Material

gkac856_Supplemental_Files
