# Supplementary material for: DAZL regulates proliferation of human primordial germ cells by direct binding to precursor miRNAs and enhances DICER processing activity
Source: Nucleic Acids Res. 2022 Oct 24;50(19):11255–72. doi: 10.1093/nar/gkac856 (PMC9638919; doi:10.1093/nar/gkac856)
Supplement: gkac856_Supplemental_Files [file gkac856_supplemental_files.zip › S. Fig legend 20220914.docx]

**SUPPLEMENTARY DATA**

SUPPLEMENTAL FIGURE LEGENDS

**Figure S1. Expressions of let-7a-5p and DAZL in hPGCLCs.**

1. Negative control of FISH/immunofluorescence staining in Figure 1A.
2. Relative expression of let-7a-5p in overexpressing DAZL (oeDAZL) and control (CTRL) cells on day2/4/6 without FACS sorting. Normalized to U6. D2 CTRL=1. Data show mean ± SD, n=3. ** P<0.01, * P<0.05 (Student’s t-test, two-sided/unpaired).
3. Relative expression of let-7a-5p in GFP+ cells gated in (D). Normalized to U6. CTRL=1. Data show mean ± SD, n=6. ** P<0.01 (Student’s t-test, two-sided/unpaired).
4. Relative expression of DAZL in CTRL and shDAZL hPGCLCs. Normalized to GAPDH. CTRL=1. Data show mean ± SD, n=6. ** P<0.01 (Student’s t-test, two-sided/unpaired).

**Figure S2. The expression levels of the mature miRNAs after DAZL silencing in PGCLCs**

1. Relative expression level (fold change) of the 118 upregulated miRNAs in the control and shDAZL PGCLCs. **** denote significant different with p-value <0.0001
2. Pie chart showing the expression trends of the 118 upregulated miRNAs.
3. Heatmap expression of the 93 significantly downregulated miRNAs after DAZL silencing.
4. Relative expression level (fold change) of the 89 downregulated miRNAs in the control and shDAZL PGCLCs. **** denote significant different with p-value <0.0001
5. Pie chart showing the expression trends of the 6 downregulated miRNAs.
6. Heatmap expression of the 62 significantly upregulated miRNAs after DAZL silencing.

**Figure S3. RNA-seq and predicted target analysis in CTRL and oeDAZL hPGCLCs**

1. Heatmap analysis showing DEG of CTRL and oeDAZL hPGCLCs.
2. Pie chart showing the expression trends of predicted gene targets of top20 upregulated or downregulated miRNAs upon DAZL overexpression.
3. Heatmap expression of the downregulated or upregulated predicted targets.
4. GO biological processes enrichment of downregulated or upregulated predicted targets.

**Figure S4. Immunostaining of human fetal ovary and calculations of adjusted pre-miRNA.**

1. Co-imunostaining of DAZL and DGCR8, and DAZL and DICER of human fetal ovary in wk10 and wk12. Scale bar, 10 μm.
2. Adjusted pre-miRNA quantifications and calculation process, showing the formula of normalized quantification of pre/pri-miRNA, normalized quantification of pri-miRNA, and adjusted pre-miRNA quantifications for let7c, miR199a, and miR10a.

**Figure S5. eCLIP-seq results of DAZL binding frequency to the top 10 upregulated miRNAs and top 10 downregulated miRNAs.** The sequencing results mapping to the locations of the indicated miRNAs were shown for the control eCLIP samples without crosslinks (NO Crosslink CTRL), input, and the DAZL eCLIP samples (DAZL CLIP).

**Figure S6. Detailed information of recombinant GST-DAZL proteins and precursor miRNAs.**

1. Coomassie blue staining and western blot of recombinant GST, GST-DAZL and GST-RRM proteins purified from E. coli for EMSA assay. Asterisk indicates the contaminant protein in purified recombinant GST-DAZL proteins. The mass spectrometry analysis of purified GST-DAZL protein showed that the main contaminant proteins (asterisk) were E. coli chaperonin, which did not influence the following assay.
2. Sequence composition of precursor miRNA probes used in this paper. Blue color indicates mature sequence, red color indicates miRNA* sequence. The sequence in bold indicates terminal loop.

**Figure S7. DAZL binds precursor miRNAs with different affinity.**

1. EMSA analysis of direct binding of purified GST, GST-DAZL and GST-RRM to FAM labeled pre-miR-199a probe.
2. EMSA analysis of direct binding of purified GST, GST-DAZL and GST-RRM to FAM labeled pre-let-7i probe.
3. Quantifications of percentage of shifted pre-miRNAs for pre-let-7i, pre-let-7c, pre-miR-199a, and pre-miR-185.
4. Fluorescence polarization (FP) experiments of FAM labeled pre-let-7c-3p/5p probes with GST-DAZL proteins. The graphs show mean ± SD (n = 3).
5. Fluorescence polarization (FP) experiments of FAM labeled pre-let-7c loop probes with GST-DAZL proteins. The graphs show mean ± SD (n = 3).

**Figure S8. DAZL binding to putative GUU motif analysis and mutation analysis.**

1. The results of precursor miRNA motif search using 118 upregulated miRNAs and 89 downregulated miRNAs.
2. EMSA analysis of direct binding of purified GST-DAZL to loop sequence of pre-let-7c with mutation besides the GUU motif.

**Figure S9. Dose dependency of DAZL on dicing activity and replicates of processing assays.**

1. Quantification of the dicing efficiency measuring the percentage of mature let-7 bands with different concentration of DAZL. Gel image is the same as in Figure 4C with higher graphical contrast to show weak bands of mature let-7.
2. Replicate experiments of Figure 4A showing reproducible results of DAZL enhancing DICER processing activity.
3. Replicate experiments of Figure 4D showing reproducible results of DAZL preferentially binding enhancing DICER processing activity more on pre-let-7 than pre-miR185.

**Figure S10. Protein purity of truncated DAZL and replicate experiments of the processing assays using truncated DAZL.**

1. Coomassie blue staining of purified GST-tagged DAZL(1-190), DAZL(1-166) and DAZL(167-190) proteins.
2. Replicate experiments of Figure 4G showing differential enhancing effect of the truncated DAZLon DICER processing activity.

**Figure S11. Predicted miRNA binding sites at the 3’UTR of TRIM71 (A) and increase of TRIM71 expression upon DAZL silencing (B).**

**Figure S12. The combination of upregulated miRNAs decreases proliferation of NT2 cells.**

1. FACS analysis of EdU incorporation of mixed miRNA overexpressing group (miR-mix) and single miRNA overexpressing group (let-7i-5p, miR-10b-5p, miR-199a-5p), and control group (LacZ) in NT2. Gating represent EdU+ population and its percentage. FITC is a reference channel for detecting the level of autofluorescence.
2. Quantification of EdU positive percentage in (A). Data show mean ± SD, n= 3. *** P<0.01 (One-way ANOVA followed by multiple comparisons with CTRL).

**Supplementary Movie 1. High resolution 3D imaging of DAZL and DICER immunostaining of human fetal ovary in wk12.**
