# Supplementary material for: DAZL regulates proliferation of human primordial germ cells by direct binding to precursor miRNAs and enhances DICER processing activity
Source: Nucleic Acids Res. 2022 Oct 24;50(19):11255–72. doi: 10.1093/nar/gkac856 (PMC9638919; doi:10.1093/nar/gkac856)
Supplement: gkac856_Supplemental_Files [file gkac856_supplemental_files.zip › S. Figure 20220912.pdf]

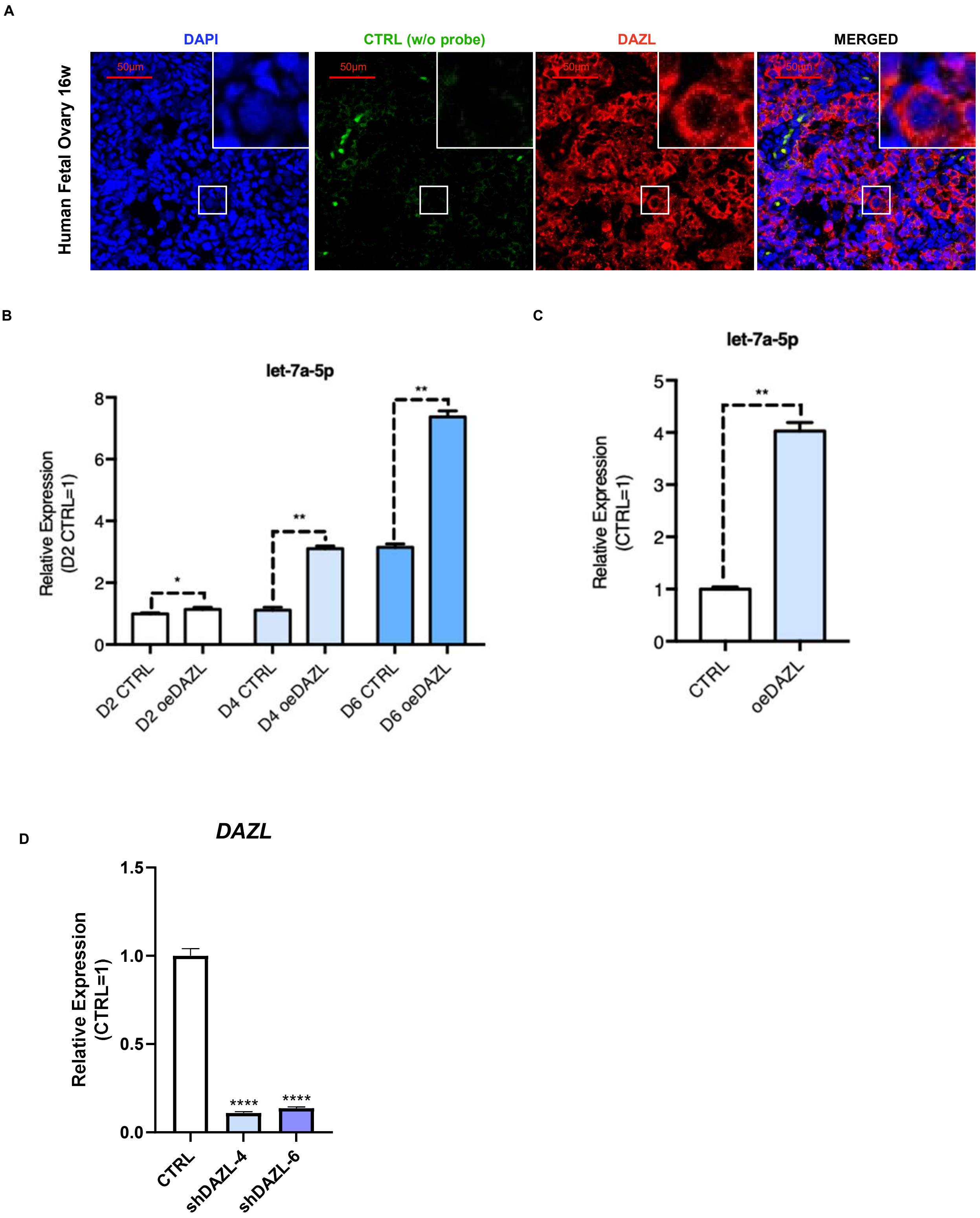

Supplementary Figure S1. Expressions of let-7a-5p and DAZL in hPGCLCs.

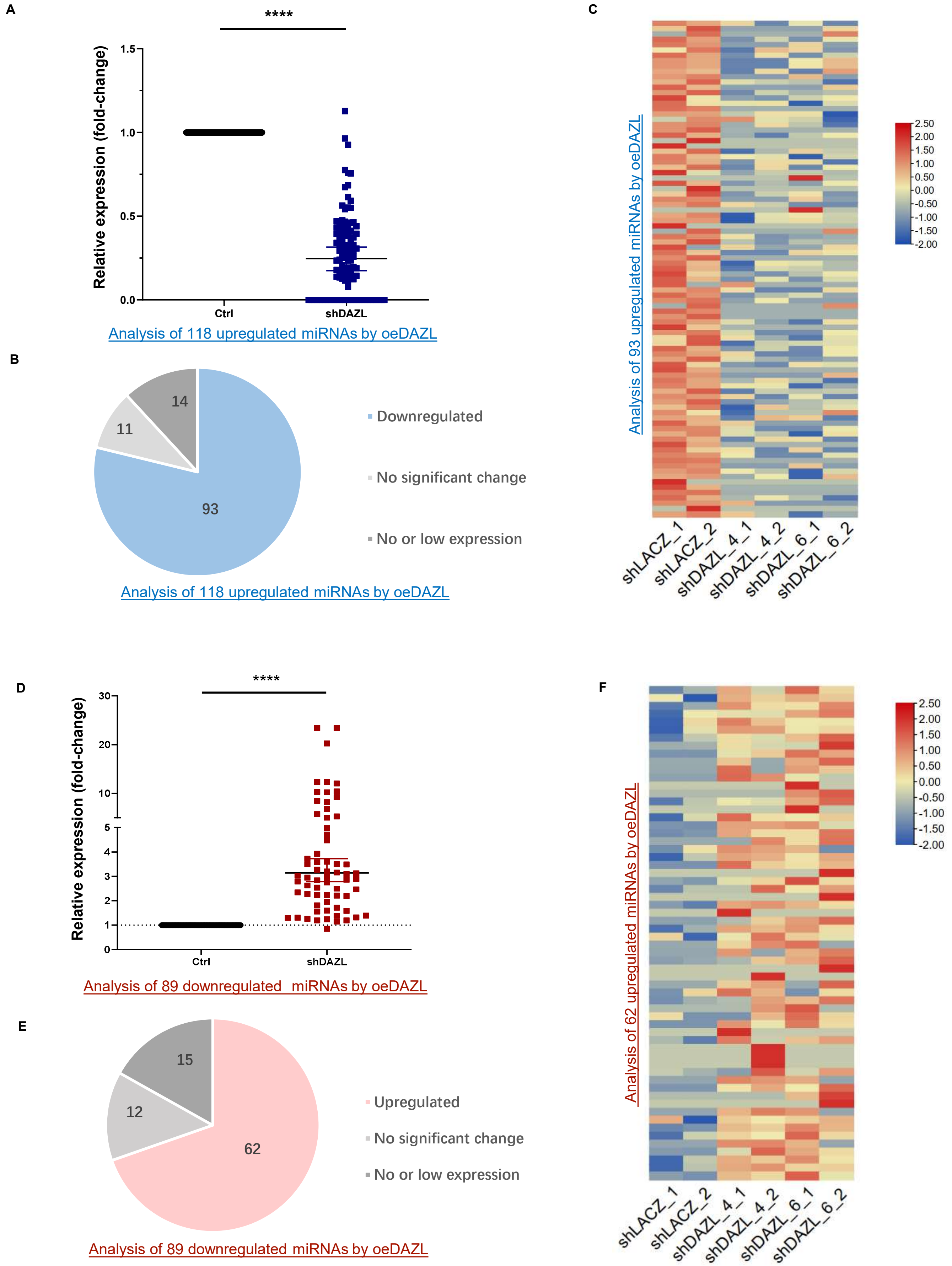

Supplementary Figure S2. The expression levels of the mature miRNAs after DAZL silencing in PGCLCs

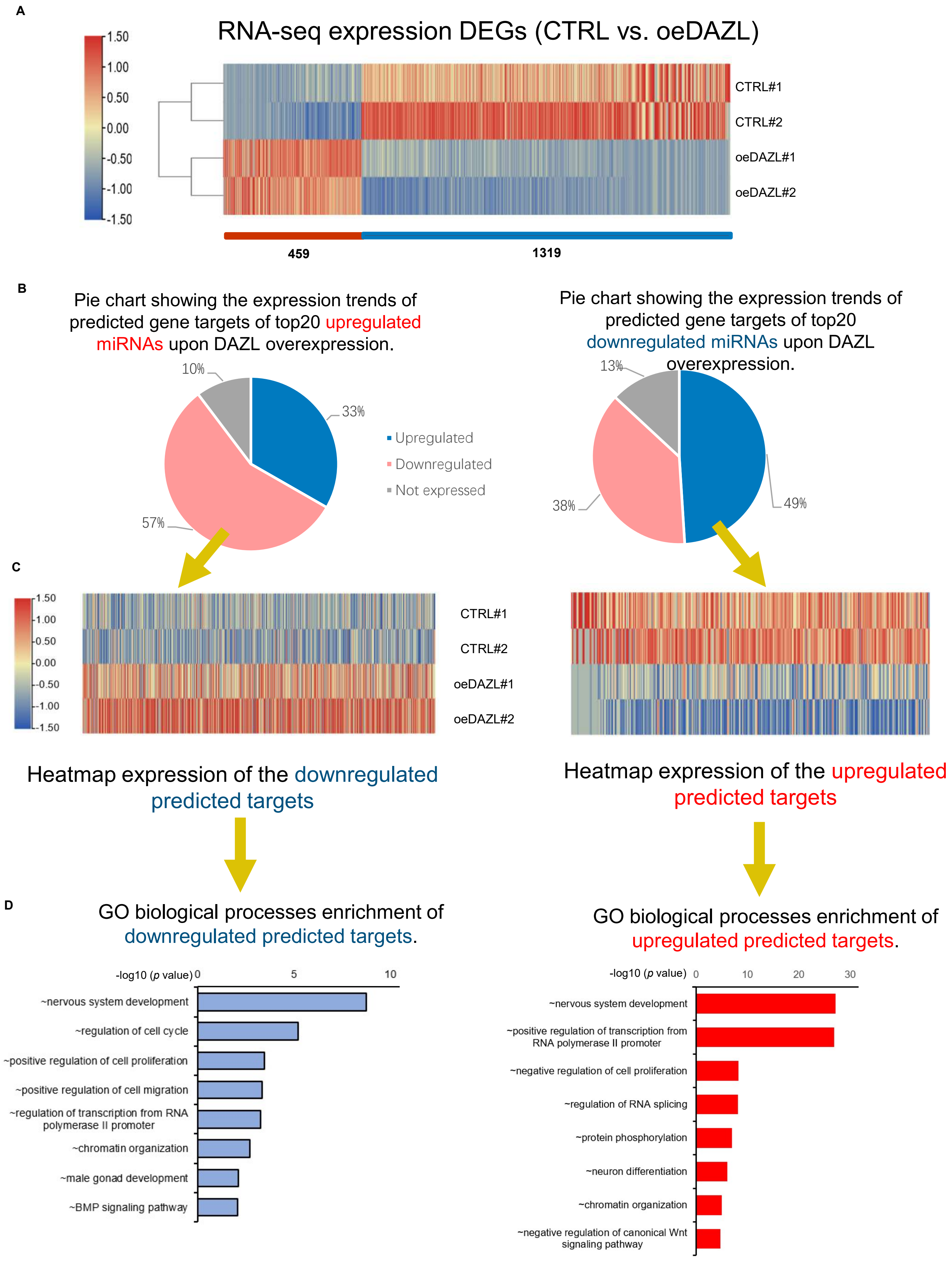

Supplementary Figure S3. RNA-seq and predicted target analysis in CTRL and oeDAZL hPGCLCs

A

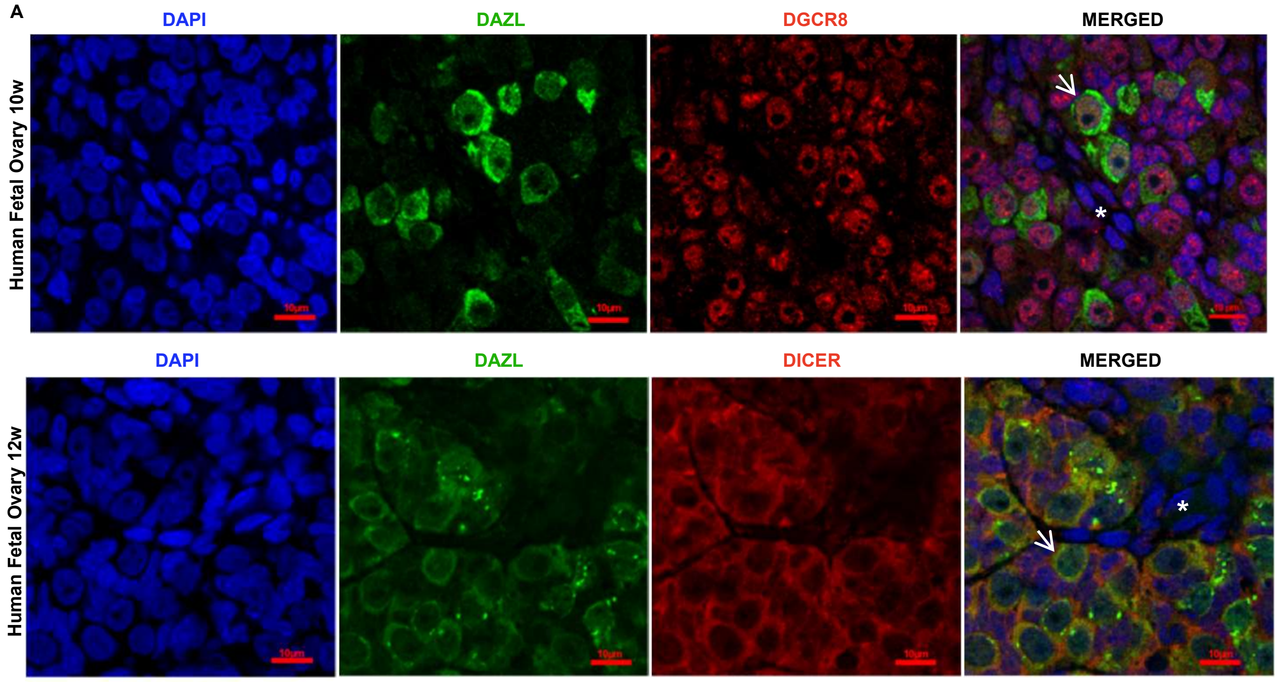

B

Adjusted pre-miRNA quantifications

- Normalized quantification of pre/pri-miRNA= 2^-[($Cq_{\text{pre+pri-miRNA(DAZL-V5 RIP)}}$ - $Cq_{\text{U6 (DAZL-V5 RIP)}}$) - ($Cq_{\text{pre+pri-miRNA(CTRL RIP)}}$ - $Cq_{\text{U6 (CTRL RIP)}}$)]
- Normalized quantification of pri-miRNA= 2^-[($Cq_{\text{pri-miRNA(DAZL-V5 RIP)}}$ - $Cq_{\text{U6 (DAZL-V5 RIP)}}$) - ($Cq_{\text{pri-miRNA(CTRL RIP)}}$ - $Cq_{\text{U6 (CTRL RIP)}}$)]
- Adjusted pre-miRNA quantifications= Normalized quantification of pre+pri-miRNA - Normalized quantification of pri-miRNA

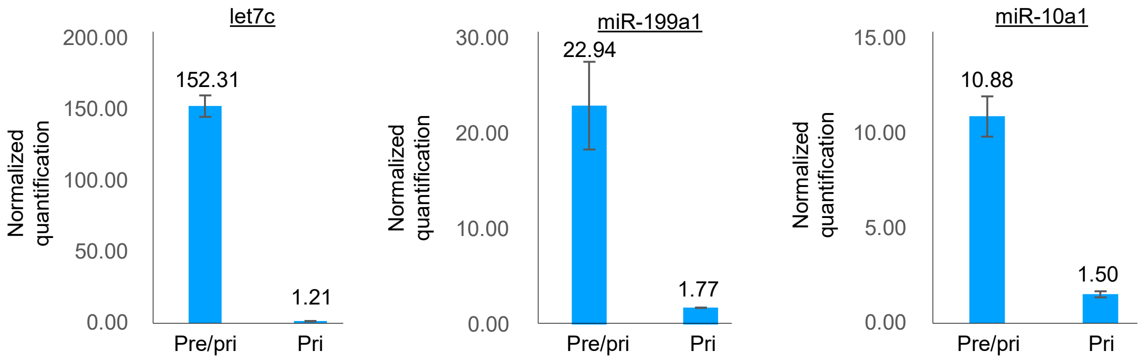

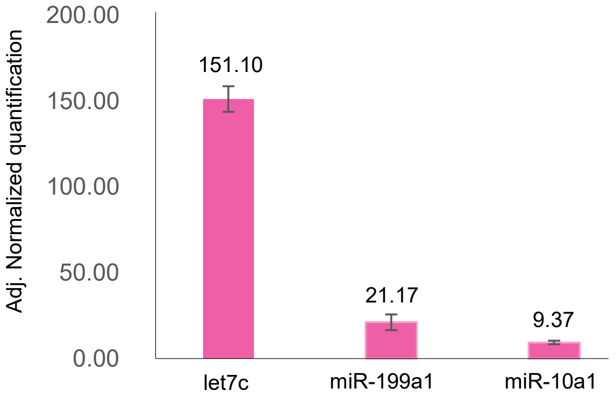

- Cq value: The PCR cycle number at the threshold line
- Error bars denote Standard deviations of technical duplicates

Supplementary Figure S4. Immunostaining of human fetal ovary and calculations of adjusted pre-miRNA.

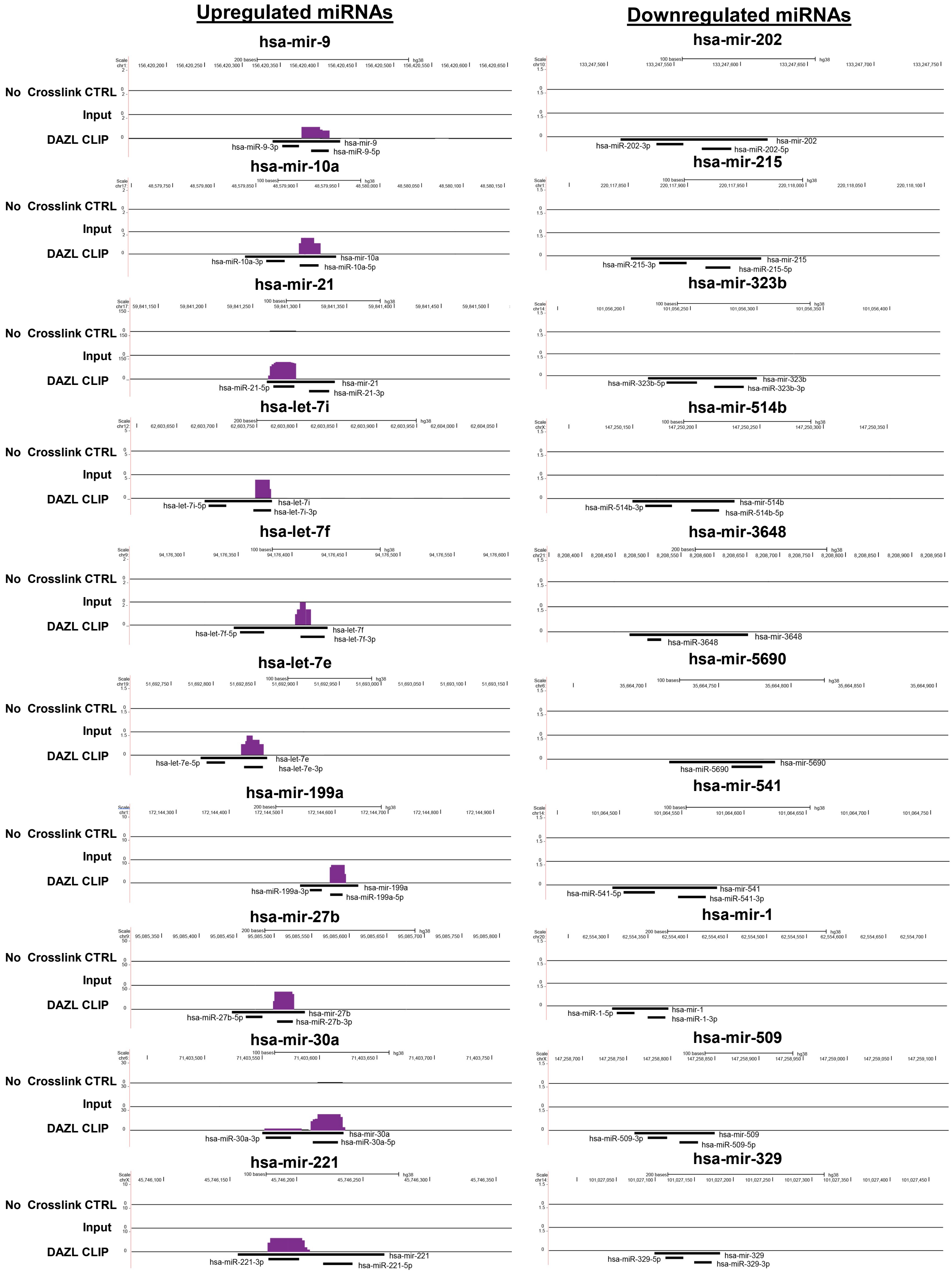

Supplementary Figure S5. eCLIP results of DAZL binding frequency to the top 10 upregulated miRNAs and top 10 downregulated miRNAs.

A

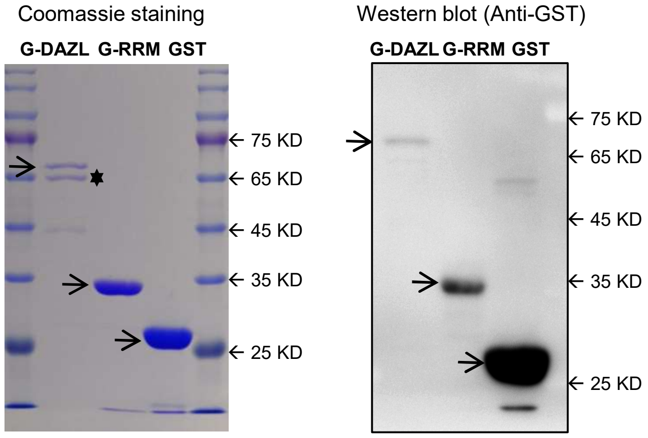

Mass spectrometric analysis of contaminant band

Ref.	Accession	Description	Score	MW [kDa]
E.coli	A0A4S4XZG3	60 kDa chaperonin OS=Escherichia coli (strain K12) OX=83333 GN=groL PE=3 SV=1	2944.33	57.3

B

let-7c stem-loop:

5'-GCAUCCGGGUUGAGGUAGUAGGUUGUAUGGUU**UAGAGUUACACCCUGGGAGUUAA**CUGUACAACCUUCUAGCUUUCCUUGGAGC-3'

let-7i stem-loop:

5'-CUGGCUGAGGUAGUAGUUUGUGCUGUU**GGUCGGGUUGUGACAUUGCCCGCUGUGGAGAUAA**CUGCGCAAGCUACUGCCUUGCUA-3'

Mature miRNA | Terminal loop | miRNA*

miR-199a1 stem-loop:

5'-GCCAACCCAGUGUUCAGACUACCUGUUC**AGGAGGCUCUCAAUGUGU**ACAGUAGUCUGCACAUUGGUUAGGC-3'

miR-185 stem-loop:

5'-AGGGGGCGAGGGAUUGGAGAGAAAGGCAGUUCCUGA**UGGUCCCCUCCCC**AGGGGCUGGCUUUCCUCUGGUCCUUCCCUCCCA-3'

Supplementary Figure S6. Detailed information of recombinant GST-DAZL proteins and precursor miRNAs.

A

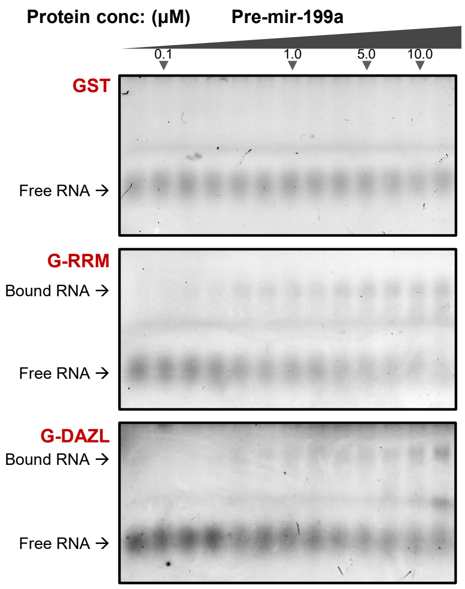

B

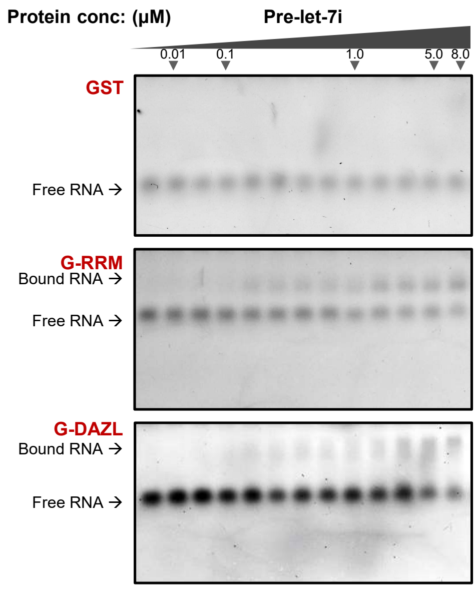

C

% Pre-miRNA shifted by G-DAZL

DAZL conc (nM)	pre-let-7i	pre-let-7c	pre-miR-199a	pre-miR-185
100	11.08%	7.24%	10.00%	5.99%
700	10.12%	12.36%	15.29%	9.25%
1000	11.83%	18.38%	20.08%	8.04%
2000	15.31%	35.10%	16.56%	6.08%
3000	36.95%	37.42%	18.15%	11.17%
5000	50.91%	41.31%	22.14%	9.81%

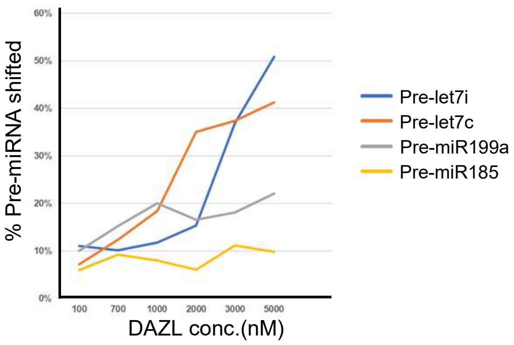

D

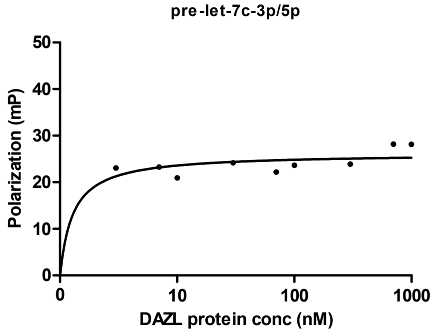

E

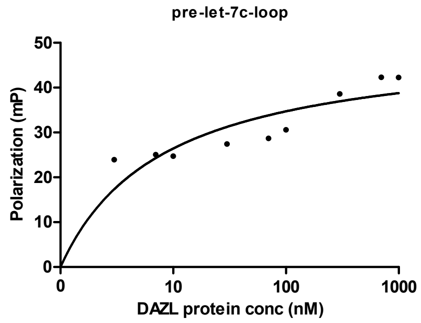

Supplementary Figure S7. DAZL binds precursor miRNAs with different affinity.

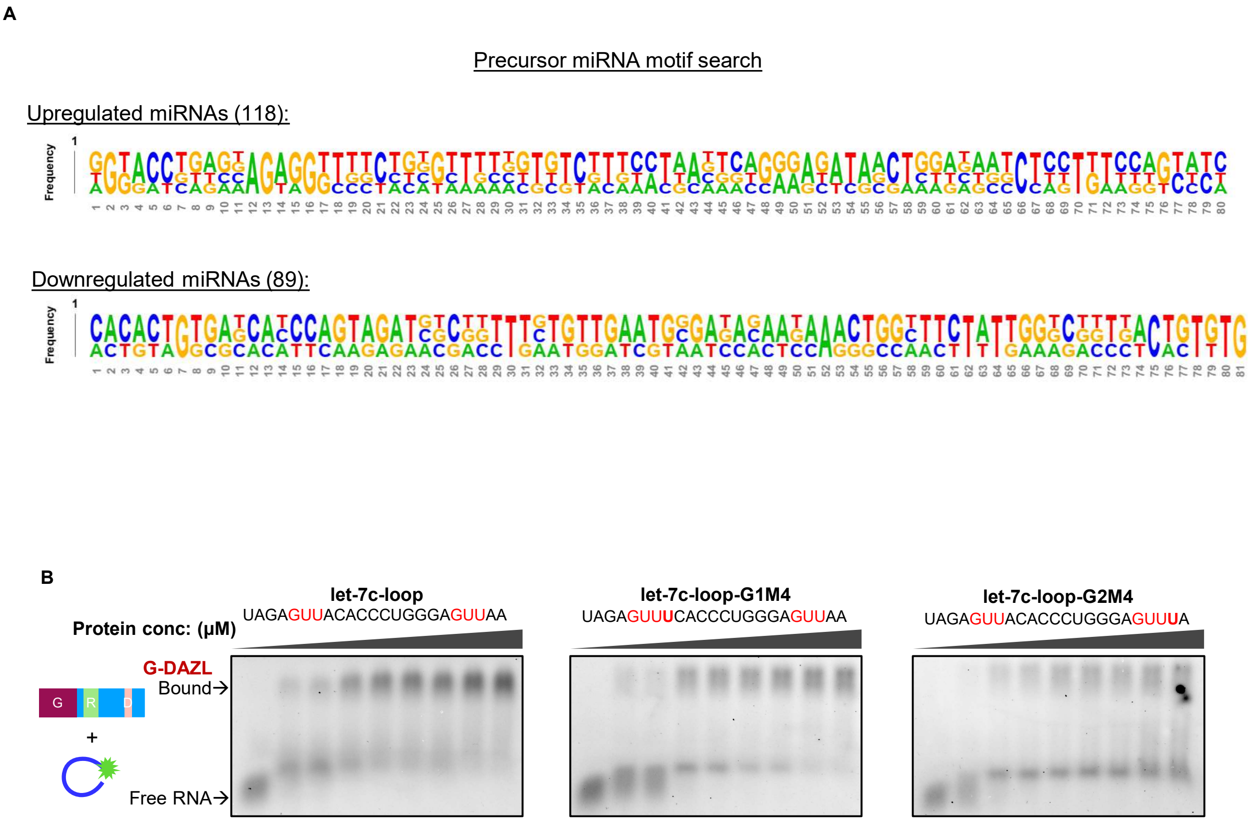

Supplementary Figure S8. DAZL binds precursor miRNAs with different affinity.

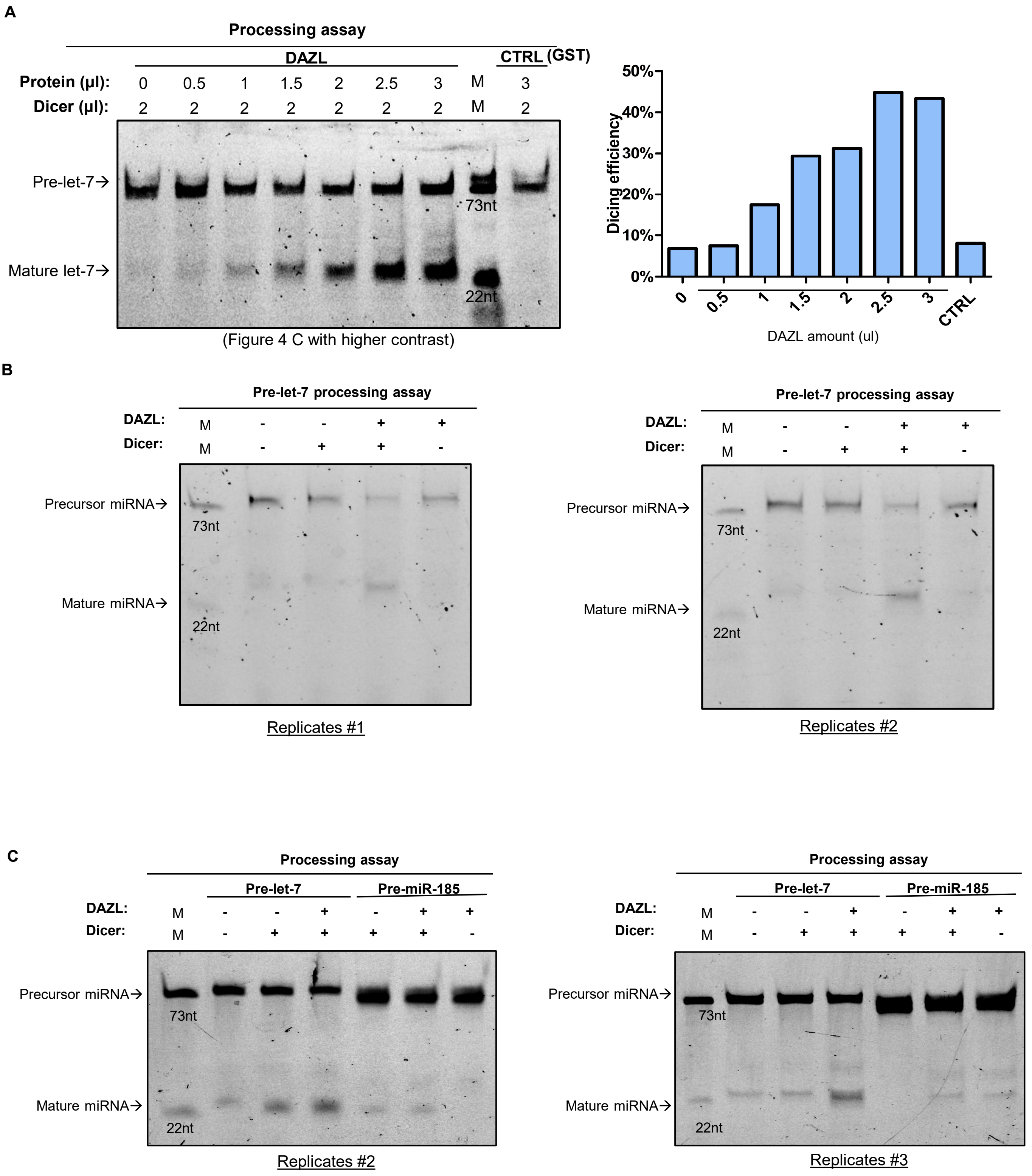

Supplementary Figure S9. A. Dose dependency of DAZL on dicing activity and replicates of processing assays.

A

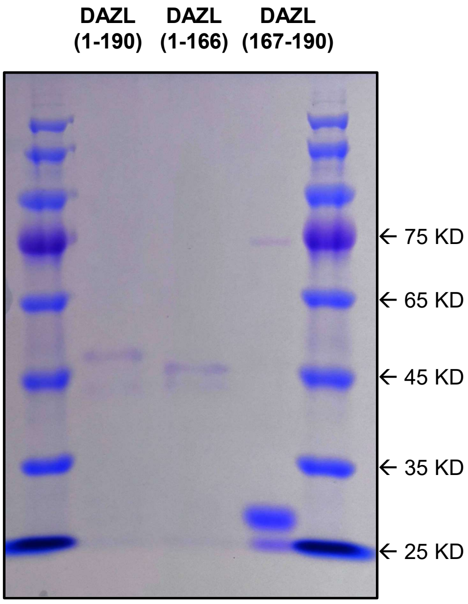

B

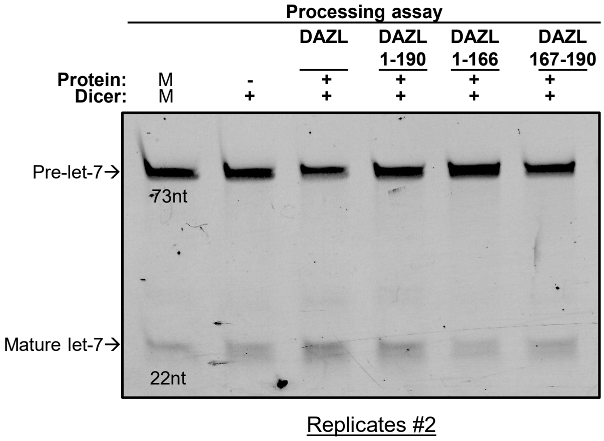

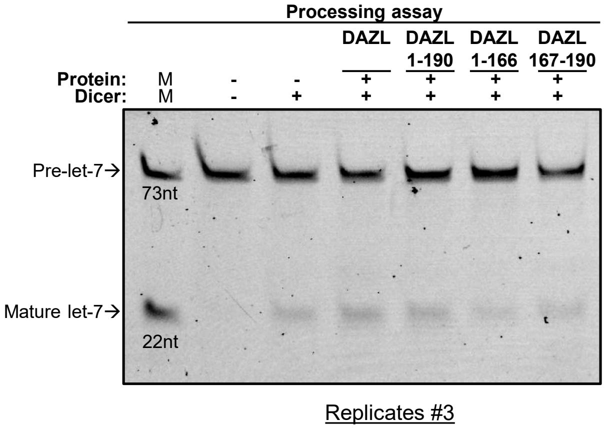

Supplementary Figure S10. Protein purity of truncated DAZL and replicate experiments of the processing assays using truncated DAZL.

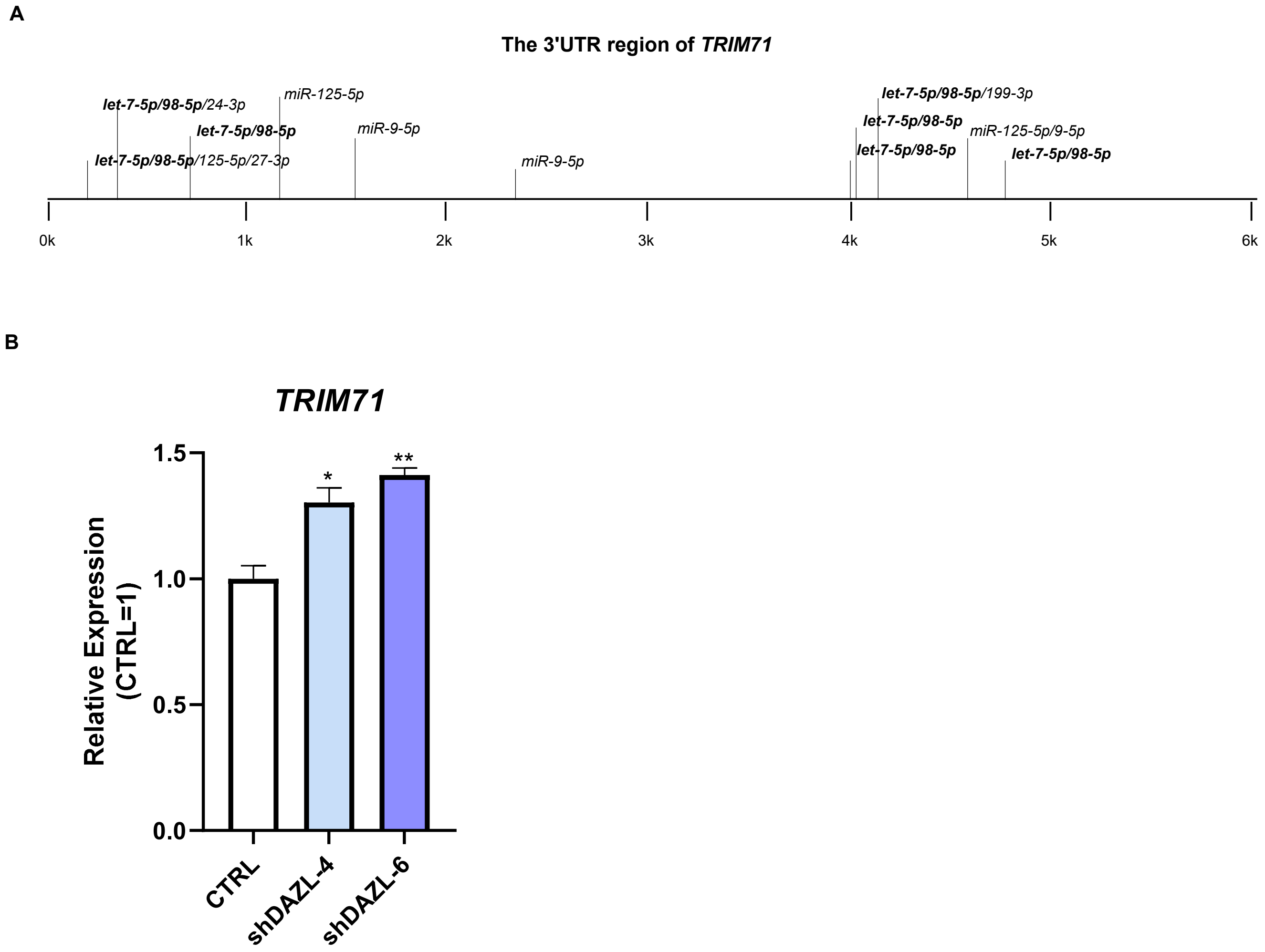

Supplementary Figure S11. Putative miRNA binding sites at the 3'UTR of TRIM71.

A

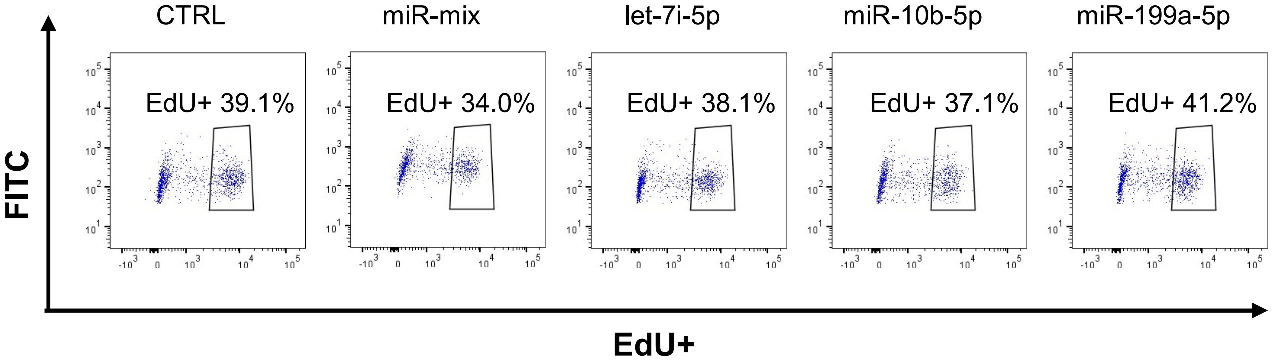

B

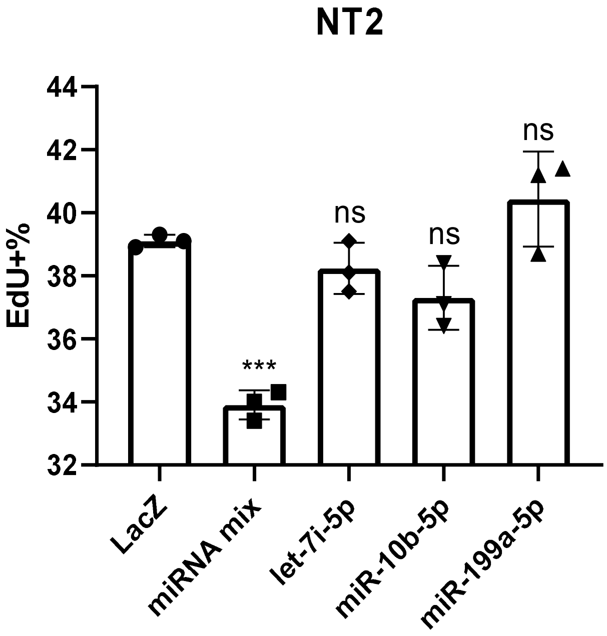

Supplementary Figure S12. The combination of upregulated miRNAs decreases proliferation of NT2 cells.
